# A Computational Probe into the Structure and Dynamics of the Full-Length Toll-Like Receptor 3 in a Phospholipid Bilayer

**DOI:** 10.3390/ijms21082857

**Published:** 2020-04-19

**Authors:** Mahesh Chandra Patra, Maria Batool, Muhammad Haseeb, Sangdun Choi

**Affiliations:** Department of Molecular Science and Technology, Ajou University, Suwon 16499, Korea; ml2mahesh@gmail.com (M.C.P.); mariabatool.28@gmail.com (M.B.); haseeb3389@hotmail.com (M.H.)

**Keywords:** Toll-like receptor 3, molecular dynamics simulation, phospholipid bilayer

## Abstract

Toll-like receptor 3 (TLR3) provides the host with antiviral defense by initiating an immune signaling cascade for the production of type I interferons. The X-ray structures of isolated TLR3 ectodomain (ECD) and transmembrane (TM) domains have been reported; however, the structure of a membrane-solvated, full-length receptor remains elusive. We investigated an all-residue TLR3 model embedded inside a phospholipid bilayer using molecular dynamics simulations. The TLR3-ECD exhibited a ~30°–35° tilt on the membrane due to the electrostatic interaction between the N-terminal subdomain and phospholipid headgroups. Although the movement of dsRNA did not affect the dimer integrity of TLR3, its sugar-phosphate backbone was slightly distorted with the orientation of the ECD. TM helices exhibited a noticeable tilt and curvature but maintained a consistent crossing angle, avoiding the hydrophobic mismatch with the bilayer. Residues from the αD helix and the CD and DE loops of the Toll/interleukin-1 receptor (TIR) domains were partially absorbed into the lower leaflet of the bilayer. We found that the previously unknown TLR3-TIR dimerization interface could be stabilized by the reciprocal contact between αC and αD helices of one subunit and the αC helix and the BB loop of the other. Overall, the present study can be helpful to understand the signaling-competent form of TLR3 in physiological environments.

## 1. Introduction

Toll-like receptor 3 (TLR3) is an important pattern recognition receptor that recognizes double-stranded RNA (dsRNA), which is a viral replication intermediate [1], and its synthetic analog polyinosinic:polycytidylic acid [2]. TLR3 is predominantly expressed in the endosomal compartment of sentinel cells such as macrophages and myeloid dendritic cells, where the recognition of endocytosed dsRNA occurs at an acidic pH (pH ≤ 6.5) [3,4]. The activated TLR3 mounts a strong immune response against invading viruses by triggering the expression of proinflammatory cytokines, typically antiviral interferons (IFNs) [5]. The agonist-mediated conformational change in the receptor initiates the recruitment of an adaptor, Toll/interleukin-1 receptor (TIR) domain-containing adaptor protein inducing IFN-β (TRIF). Subsequent stages of the signaling cascade involve the recruitment of the inhibitor of nuclear factor-κB-kinase-ε (IKK-ε) complex and the phosphorylation of TANK-binding kinase 1 (TBK1) [6]. The phosphorylated TBK1 activates the transcription factor IFN-regulatory factor 3 (IRF3), which induces the expression of type I IFNs (IFN-α/β) [7]. In addition, dsRNA-mediated TLR3 signaling leads to the activation of a number of transcription factors, specifically nuclear factor kappa-light-chain-enhancer of activated B cells (NF-κB), IRF-7, and members of the activator protein 1 (AP-1) family [8].

TLR3 exhibits a tripartite domain architecture, comprising a leucine-rich repeat (LRR)-rich ectodomain (ECD), a transmembrane (TM) domain, and a cytosolic Toll/interleukin-1 receptor (TIR) domain [9]. The structure of mouse TLR3-ECD complexed with dsRNA has been resolved through X-ray crystallography [10], and the nuclear magnetic resonance (NMR) structure of the TM domain in its dimeric and trimeric forms have been reported [11]. However, the structure of the TIR domain or the full-length, intact TLR3 structure has remained elusive. Phylogenetic analysis has categorized vertebrate TLRs into different subfamilies, including TLR1/2/6/10, TLR3, TLR4, TLR5, TLR7/8/9, and TLR11/12/13/21/22/23, suggesting that TLR3 belongs to a unique category within the TLR family [12]. Because TLR3 triggers a strong immune response against viral infection, it is considered a promising drug target for the development of effective vaccine adjuvants [13]. The dsRNA-specificity of TLR3 is independent of nucleotide composition; therefore, a large number of potent adjuvants has been developed and investigated in clinical trials [14,15]. Although TLR3 signaling is crucial for the protection of the host against viral infections [16], dysregulated signaling has been implicated in several inflammatory disorders [17], autoimmune diseases [18], cancer [19], and atherosclerosis [20]. Synthetic monoclonal antibodies [21,22] and low-molecular-weight compounds [23,24] have shown beneficial effects on TLR3-mediated diseases in mice and human subjects [25].

Despite the clinical significance of TLR3 modulation, the structural organization of full-length TLR3 in an activated state has not been experimentally determined, probably due to its complex architecture that spans the membrane and the aqueous environment of the cell. Earlier, Liu et al. (2008) have proposed a full-length model of TLR3 [10]; however, the atomistic details of inter-domain interactions, time-dependent structural evolution, and dynamics of TM, TIR, and ECD with respect to the phospholipid bilayer as a single unit have not been thoroughly studied. In the present study, we analyzed a full-length homodimerization complex of TLR3 embedded in a 1-palmitoyl-2-oleoyl-sn-glycero-3-phosphocholine (POPC) bilayer using molecular dynamics (MD) simulation techniques. The membrane-solvated TLR3 model was able to elucidate several important structural features of the individual ECD, TM, and TIR domains in a physiologically applicable environment. The ECD and TIR domains are established drug targets; therefore, a detailed knowledge about their structural topology and intermolecular interactions is crucial for the development of effective, novel adjuvant, or antagonistic candidates.

## 2. Results

### 2.1. Full-length TLR3 Tilts and Wraps around the Phospholipid Bilayer

**Figure 1:**
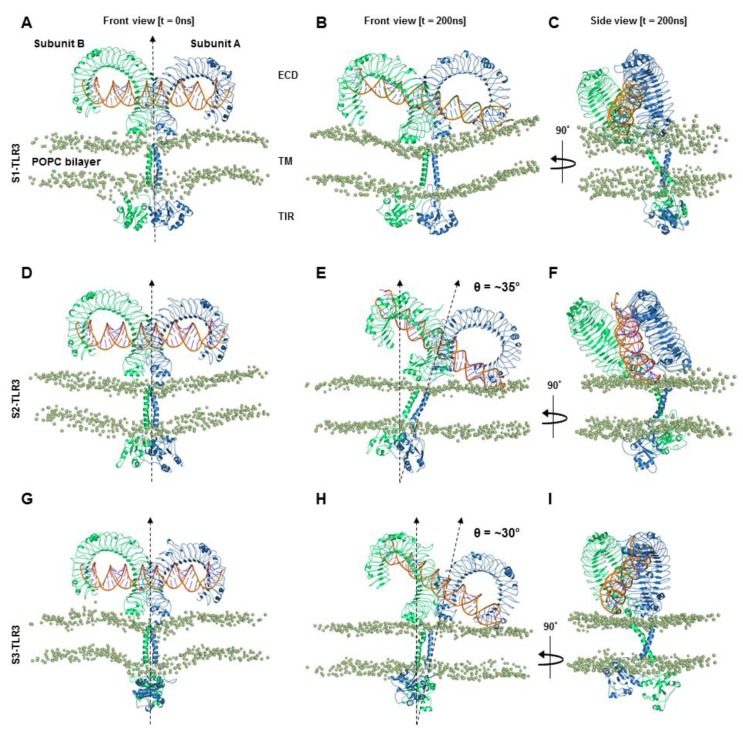
Overall structure of Toll-like receptor (TLR3) in 1-palmitoyl-2-oleoyl-sn-glycero-3-phosphocholine (POPC) bilayers. (**A**) Initial structure of S1-TLR3 (0 ns). (**B**) Final snapshot structure of S1-TLR3 (200 ns). (**C**) Side view of panel B (90° rotation). (**D**) Initial structure of S2-TLR3. (**E**) Final snapshot structure of S2-TLR3. (**F**) Side view of panel E. (**G**) Initial structure of S3-TLR3. (**H**) Final snapshot structure of S3-TLR3. (**I**) Side view of panel G. Subunits A and B are colored blue and green, respectively. The phosphate atoms of the bilayer lipids are shown as mauve beads. S1, S2, and S3 represent replicate 2 of simulation set 1, 2, and 3, respectively. ECD, extracellular domain; TM, transmembrane domain; TIR, Toll/interleukin receptor domain; TLR3, Toll-like receptor 3; POPC, 1-palmitoyl-2-oleoyl-sn-glycero-3-phosphocholine; t, simulation time; θ, tilt angle of ECD with respect to the bilayer normal.

To establish a molecular basis for the complete structural organization of TLR3, we constructed three separate models of the full-length TLR3-dsRNA homodimerization complex and simulated them in POPC bilayers for a duration of 200 ns. Each MD simulation was based on a distinct TLR3 model, where the TIR domains of S1-, S2-, and S3-TLR3 were constructed from those of TLR2 [26], TLR6 [27], and TLR10 [28], respectively (Supplementary material, Table S1). Stereochemical accuracy of the starting models are shown in Supplementary material, Table S2. Three independent simulations were carried out for each TLR3-POPC system by assigning random initial velocity during the equilibration phase (Supplementary material, Table S3). Superimposition of low-energy average coordinates extracted from individual trajectories of each set showed structural convergence (Supplementary material, Figure S1). In the MD simulations, a minor conformational change was observed in S1-ECD (Figure 1A–C), while S2- and S3-TLR3 exhibited considerable orientation and structural changes within the membrane bilayer. Specifically, S2- and S3-ECD underwent a progressive tilt with reference to the bilayer normal (i.e., the Z-axis) due to the contact of their N-terminal subdomain (LRR-NT; residues 24–51) and LRRs 1-3 (residues 52–121) with the polar headgroups of the bilayer (Figure 1D–I). While S1-ECD tilted slightly on its forward face (Figure 1C), S2- and S3-ECD tilted ~30°–35° in a lateral direction along with a forward tilt (Figure 1E,H).

**Figure 2:**
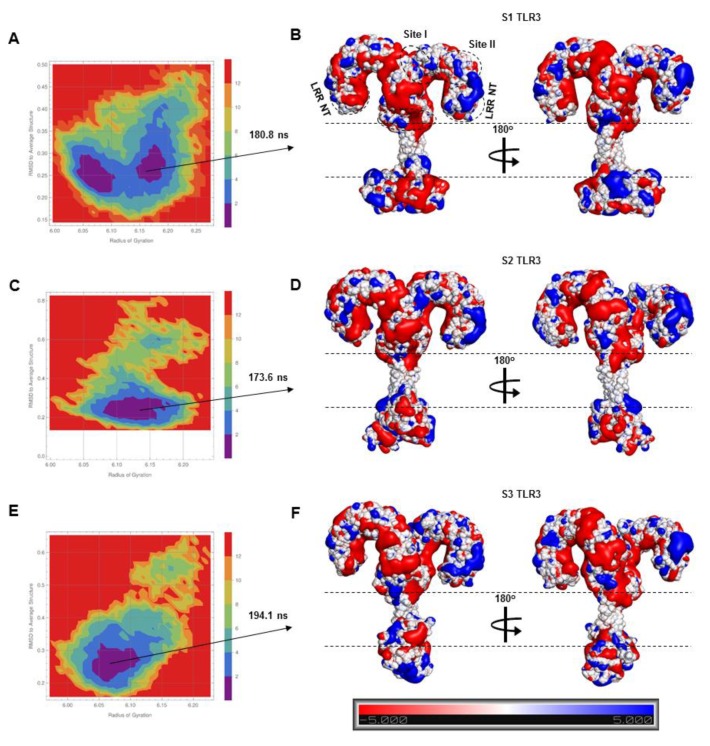
The Gibbs free energy landscape (FEL) and electrostatic properties of TLR3. (**A**,**C**,**E**) The FEL of S1, S2, and S3-TLR3 sampled across the molecular dynamics (MD) trajectories of replicate 2. The blue region in the center of the plot indicates the low-energy conformations of the protein. The electrostatic potential surfaces were constructed from a representative conformation extracted from the low-energy structural ensemble of each FEL. (**B**,**D**,**F**) The electrostatic potential surface of the TLR3 homodimer. Blue, red, and white colors represent positively charged, negatively charged, and neutral surfaces, respectively. A 180° view of each model is shown alongside of each panel. Dashed lines indicate the approximated membrane boundary.

To understand the tilting behavior of ECDs over the membrane, we calculated the electrostatic potential surfaces of representative low-energy coordinates of TLR3 from the Gibbs free energy landscape (Figure 2A,C,E). The TLR3-ECDs exhibited distinct acidic and basic surface patches at the LRR-NT and LRRs 1-3 subdomains (Figure 2B,D,F), which contain an equal number of positively charged (R + K = 9 (9.2%)) and negatively charged (D + E = 9 (9.2%)) residues, along with several aliphatic (L = 17 (17.3%)) and relatively polar (T = 11 (11.2%)) residues. The side chains of these amino acids could form strong electrostatic and van der Waals contact with the zwitterionic phospholipid headgroups, thus stabilizing the receptor on the membrane. The major difference in the distribution of the electrostatic potential surfaces on the three TLR3 models occurs at the TIR domains because of their distinct topological organization.

The sharp movement of ECD exerted a minor fluctuation in the local helical geometry of dsRNA backbone over the course of the simulation (Supplementary material, Figure S2A–F), while the dsRNA remained tightly bound to the two positively charged binding sites, i.e., site I and site II, in the ECD. Calculation of the root mean square deviation (RMSD) as a function of simulation time revealed that the S1-dsRNA backbone became rather flexible after ~120 ns in all three replicates (Supplementary material, Figure S2G); however, the backbone RMSDs of S2- and S3-dsRNA were stable throughout the simulation (Supplementary material, Figure S2H,I). The orientation of the ECD on the membrane was accompanied by the tilting action of the TM domains, crossing each other at specific angles in all three sets of simulations. TM helices normally tilt or bend due to a hydrophobic mismatch with the bilayer core, and this phenomenon has been observed in the TM peptides of TLR4 [29]. The snorkeling of the membrane-facing TIR residues around the phospholipid headgroups was observed before they were partially absorbed into the lower surface of the bilayer. We speculate that the distinct interface of each TLR3-TIR dimer may have influenced the specific orientation of the upstream domains during the MD simulations.

### 2.2. The S2-TLR3-POPC System Maintains Better Stability During MD Simulations

**Figure 3:**
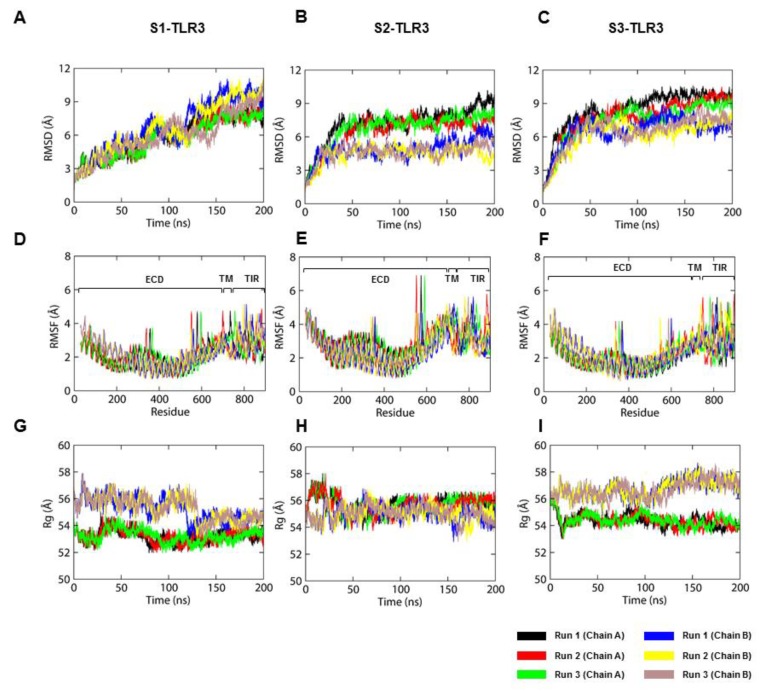
Stability parameters of the three TLR3 models over the course of the simulation. (**A**–**C**) Root mean square deviation. (**D**–**F**) Root mean square fluctuation. (**G**–**I**) Radius of gyration. The black and red lines represent subunits A and B, respectively.

In the analysis of RMSD as a function of simulation time, we found that the backbone atoms (N-Cα-C) of each TLR3 model showed distinct behavior during MD simulations. The backbone RMSDs of the three replicates of S1-TLR3 did not converge until 200 ns but those of S2- and S3-TLR3 reached the equilibrium plateau soon after 50-ns MD simulation (Figure 3A–C). RMSD analyses of individual domains revealed that chain B of S2-ECD (blue, yellow, and brown) deviated greater than that of S1- or S3-ECDs in all three replicates (Supplementary material, Figure S3). Except ECDs, the dynamics of TM and TIR domains of all three models were largely consistent throughout the MD simulations. This indicates that the differential RMSD patterns of the whole protein compared to the individual domains is most likely due to the long, flexible juxtamembrane loops, which connect the TM with the ECD and TIR domains. The Cα root mean square fluctuation (RMSF) indicated that the ECD and TM domains were highly stable in all simulations while the TIR residues were rather flexible (Figure 3D–F). Radius-of-gyration (Rg) plots showed that both TLR3 subunits had distinct Rg values in all simulations, suggesting their different shape and size between complexes (Figure 3G–I). Above all, S2-TLR3 displayed relatively more compact and organized molecular architecture, with both of its subunits attaining equivalent Rg values in the equilibrium plateau of MD trajectories. The POPC bilayers were stable in all MD simulations, exhibiting acceptable biophysical parameters, such as density, thickness [30], lipid order [31], and area per lipid [32] properties (Supplementary material, Figure S4 and Table S4).

### 2.3. The S2-TLR3-dsRNA Complex Forms Relatively Steady Intermolecular Contacts within the Phospholipid Bilayer

After observing the considerable orientation and structural transition of TLR3-ECDs in phospholipid bilayers, we sought to confirm the dsRNA-binding patterns of the three MD trajectories. First, the binding free energy (BFE) of each TLR3-dsRNA complex was calculated via the molecular mechanics/Poisson–Boltzmann surface area (MM/PBSA) method in the gas phase (Table 1). Separate calculations were performed for each subunit to better distinguish the relative affinity of the protein for dsRNA. Results indicated that subunit A of TLR3 had greater affinity towards dsRNA compared to subunit B. Cumulatively, S1- and S2-TLR3-ECD bound to dsRNA stronger than S3-TLR3 did. The decomposition of BFE into individual energy components revealed that S1- and S2-TLR3-dsRNA complexes exhibit greater binding affinities as compared to S3-TLR3-dsRNA complex, owing to the contribution of electrostatic energies. An analysis of intermolecular hydrogen bonds (H-bonds) revealed that site I residues formed a comparatively greater number of H-bonds with the 2’-hydroxyl groups of dsRNA as compared to site II (Supplementary material, Table S5–S7). The H-bonds remained largely consistent with good % occupancy through most part of the simulations. During MD simulations, salt bridges or electrostatic interactions replaced some of the site II H-bonds, probably due to the tilting behavior of ECDs on the membrane. Closer analysis revealed that S3-TLR3 formed the weakest H-bond contact with dsRNA at site II, with only H39 and E110 involved in H-bond formation. Nonetheless, the secondary structures of the three TLR3-ECDs were consistent and the bound dsRNA retained a typical A-DNA-like conformation (Supplementary material, Table S8), suggesting that the overall structure of TLR3 does not change after dsRNA binding [10].

**Figure 4:**
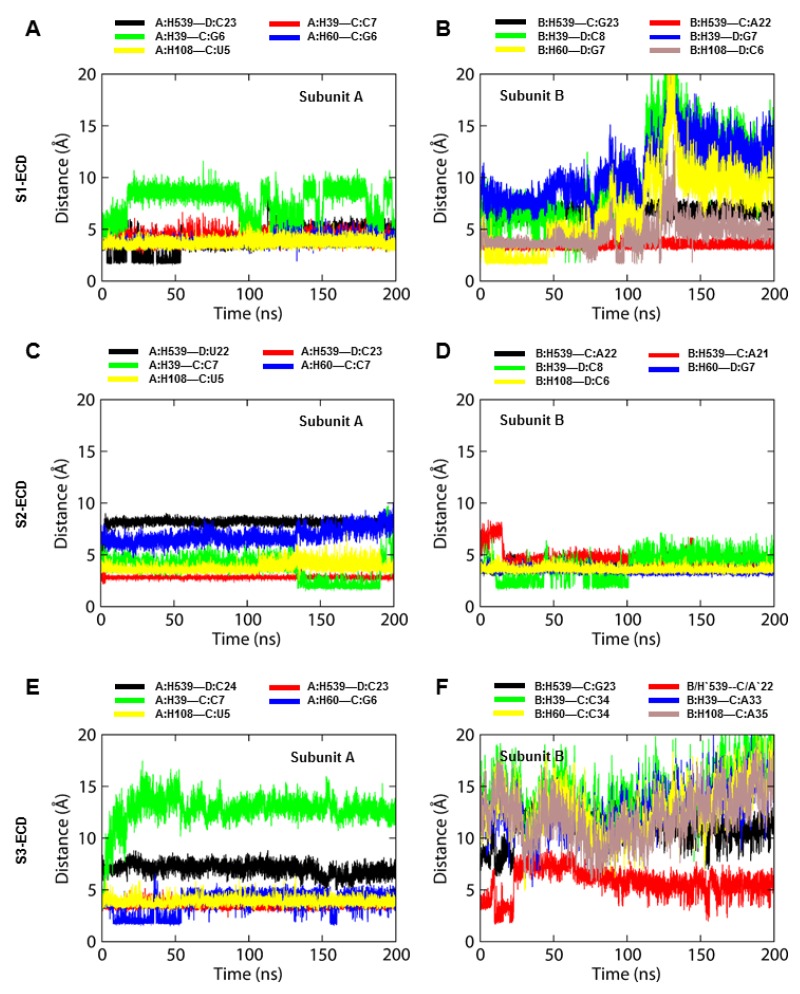
The distance between the key histidine residues of TLR3 and the phosphate groups of the dsRNA backbone. (**A**–**F**) Intermolecular distances between the imidazole rings of H539, H39, H60, and H108 and the phosphate groups of the dsRNA backbone in three MD simulation systems (replicate 2) as a function of time. Distances were calculated for both chains of the TLR3-dsRNA complex. S1, S2, and S3 represent replicate 2 of sets 1, 2, and 3, respectively.

Residues H39, H60, H108, and H539 have been reported to form salt bridges with the phosphate group of dsRNA, and alanine substitution at H39 or H60 (site I) blocked receptor activity, suggesting that these amino acids are essential for ligand recognition [9]. To assess the structural integrity of TLR3 and the evolution of dsRNA-binding patterns in the full-length models, we measured the distances between the imidazole rings of H39, H60, H108, and H539 and the phosphate groups of dsRNA as a function of simulation time. H39 was the most flexible residue in S1- and S3-TLR3, but it formed a more stable and consistent interaction with dsRNA in S2-TLR3 (Figure 4). While H60 deviated to a greater distance from dsRNA in S1- and S3-TLR3, it maintained a consistent intermolecular distance with the dsRNA backbone in S2-TLR3. The average distances between the interacting atoms in H60 and the dsRNA bases in subunits A and B were calculated to be 6.83 ± 0.73 and 3.48 ± 0.24 Å, respectively (Table 2). Similarly, residues H108 and H539 of both subunits in S2-TLR3 continued to oscillate near the phosphate groups of dsRNA over the course of the simulation, unlike those of S1- and S3-TLR3. Altogether, we found that all four key histidine residues in S2-TLR3 displayed relatively stable intermolecular distances, thus exhibiting a stronger interaction with dsRNA bound to the full-length receptor.

**Figure 5:**
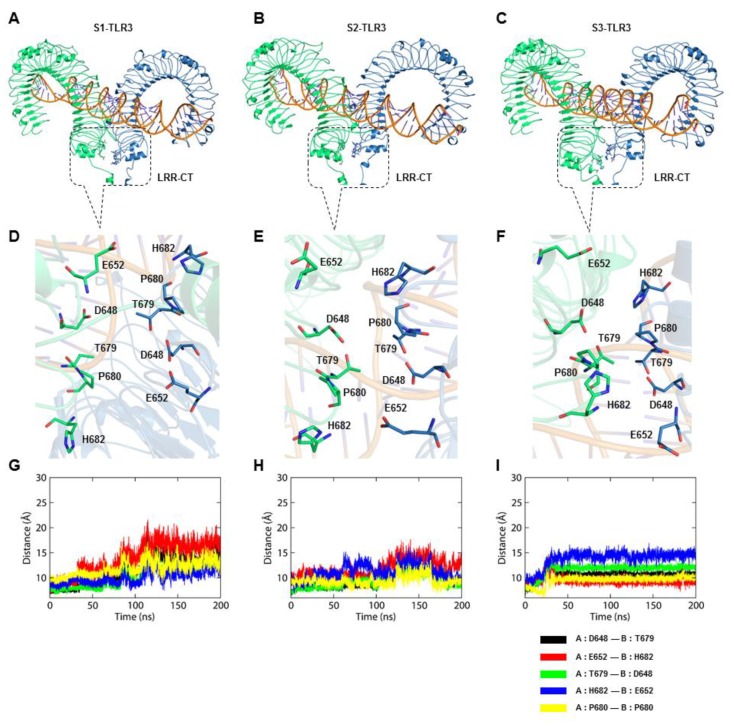
The homodimerization interface of TLR3 in the three representative MD simulation systems. (**A**–**C**) The overall structure of TLR3-ECD, showing receptor dimerization at the interface of the C-terminal subdomains. (**D**–**F**) Close-up view of amino acid orientations at the homodimerization interface of S1-, S2-, and S3-TLR3. (**G**–**I**) Distance between the chiral carbon atoms of interfacing residues as a function of simulation time. S1, S2, and S3 represent second replicate of sets 1, 2, and 3, respectively. Subunits A and B of TLR3 are colored blue and green, respectively.

The X-ray structure of mouse TLR3-dsRNA complex (PDB ID: 3CIY [10]) indicated that C-terminal subdomains (LRR-CT; residues 645–698) mediate the receptor homodimerization through a series of protein–protein interactions, facilitating the TIR domain association in the cytosol (Figure 5A–C). The intermolecular contact between the LRR-CT domains involves two H-bond pairs, D648/T679 and E652/H682, and the structural proline P680 [33]. In our MD simulations, the D648/T679 and E652/H682 H-bond pairs were transient and often substituted by electrostatic interactions as the distance between the interacting atoms increased over time (Figure 5D–I). The distance between the chiral carbon (Cα) atoms of the interfacing residues in S1-TLR3 increased with simulation time, indicating the loss of dimer integrity (Figure 5D,G). On the other hand, the LRR-CT domains of S2- and S3-TLR3 maintained reasonable proximity and formed relatively stable inter-residue contact over the entire simulation (Figure 5E–I). However, the average distance between the center of mass of interacting residues throughout the trajectory indicated that the ECD of S2-TLR3 maintained a relatively compact dimer packing interaction compared to others (Supplementary material, Table S9). The binding affinity and interaction data together suggest that the S2-TLR3-dsRNA complex better represents the activated receptor within the endosomal membrane.

### 2.4. The Hydrophobic Mismatch Affects the Dynamics of TM Domains in the Membrane-Bound TLR3

**Figure 6:**
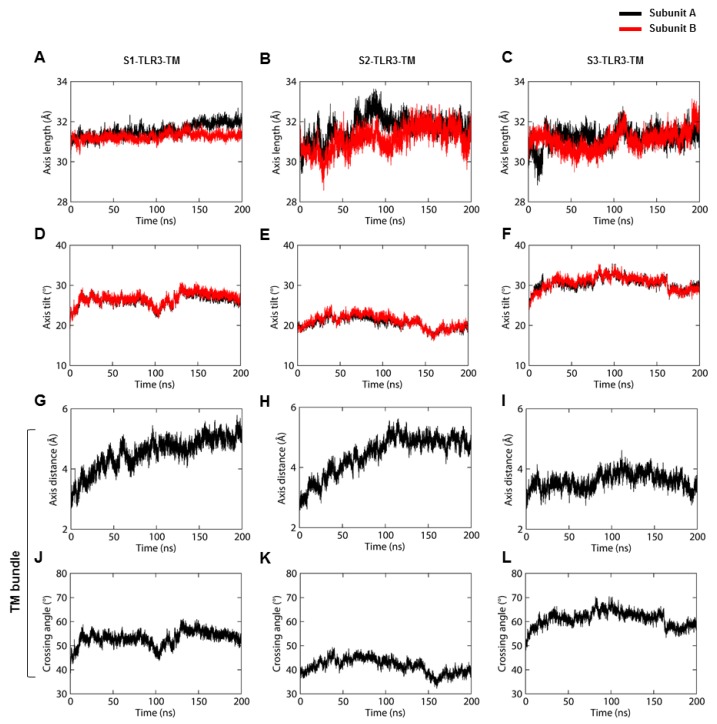
Helical properties of TM domains. (**A**–**C**) The length of the helical axis of the TM dimer. (**D**–**F**) The tilt angle of the TM helices with respect to the average angle of the helical axis. Subunits A and B are shown as black and red lines, respectively. (**G**–**I**) The distance between axis centers of individual TM domains. (**J**–**L**) Crossing angle of TM helices as a function of time. S1, S2, and S3 represent the second replicate of sets 1, 2, and 3, respectively.

To address the dynamic behavior of the TM domains in full-length TLR3, we analyzed the helical properties of TM bundles in three representative MD trajectories. The three TLR3-TM bundles exhibited different lengths and tilt angles with respect to their average helical axis. The average helical length of all TM domains remained around 31 Å, indicating no major change in the α-helicity during the dynamic condition (Figure 6A–C). While the individual helix lengths within S1- and S3-TM domains were mostly correlated, that of S2-TM elevated around 100 ns. We observed a consistent tilt angles for both subunits of the TM domains (Figure 6D–F). The average tilt of S2-TM was 20.63° in subunit A and 21.02° in subunit B. On the other hand, both subunits of S1- and S3-TM domains tilted approximately 26° and 30°, respectively (Supplementary material, Table S10). The difference in the length or tilt of the TM bundles could be explained by the irregular distance between their axis centers (Figure 6G–I). The helical axes of S2-TM maintained a greater stability after 100-ns MD simulation as opposed to S1- and S3-TM. The tilt and curvature of TM bundles were accompanied by different crossing angles, where S2-TM cross each other at an average angle of 41.66° (Figure 6J–L). The individual TM helices exhibited a per-residue twist angle of ~100 degrees, representing ideal α-helices. The variations in the helical properties of the three TM domains inside the phospholipid bilayer could be the result of the differential dynamics of the upstream ECD and downstream TIR domains together with the hydrophobic mismatch with the bilayer core. Based on the calculated values of axis length, tilt, distance, and crossing angle, S2-TM represents the most stable configuration of full-length TLR3.

**Figure 7:**
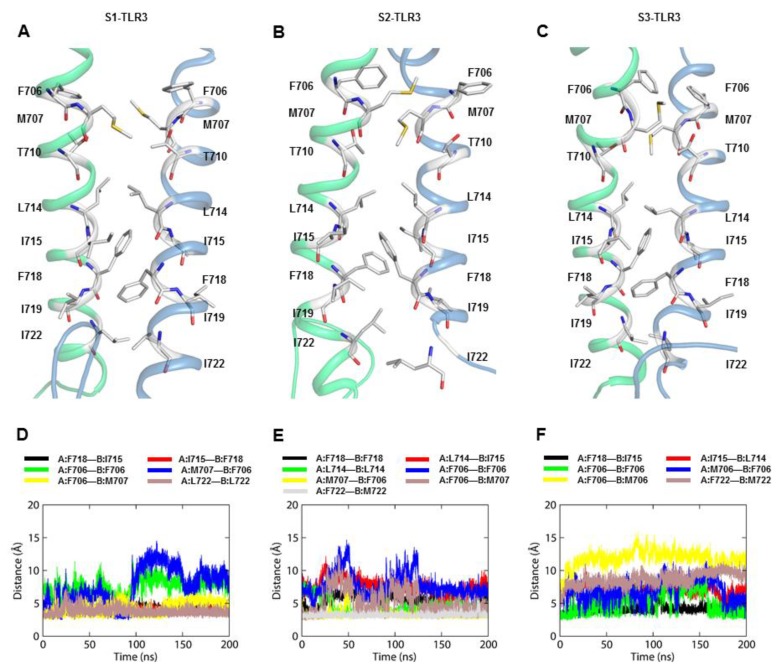
Dimer packing interaction of TM domains. (**A**–**C**) Comparison of the dimer packing interaction at the helix-helix interfaces of the S1-, S2-, and S3-TM dimers from representative low-energy conformations. Residues previously reported to play an important role in TM dimerization are highlighted as stick models. Subunit A and subunit B of the TM dimer are colored blue and green, respectively. (**D**–**F**) Time-dependent distances between the amino acid side-chains that were involved in the helix-helix interaction along the molecular dynamics trajectory. S1, S2, and S3 represent replicate 2 of sets 1, 2, and 3, respectively.

Analysis of the NMR structure of an isolated TLR3-TM dimer (D698–I730 at pH 4.5) has demonstrated that residues F706, T710, L714, I715, F718, and I719 constitute the core intermolecular contacts through reciprocal interactions [11]. The actual TM segment extends from F705 to F725, including 6 isoleucine (28.6%), 4 leucine (19.0%), and 5 phenylalanine (23.8%) and a cluster of consecutive, relatively polar amino acids (N709, T710, and S711). We compared the consistency of key inter-helical contacts throughout the three MD trajectories. The aromatic stacking of F706 (observed in the NMR structure of TLR3-TM) was displaced in S1- and S3-TM, while this interaction was preserved in the S2-TM between 100- and 200-ns MD simulations. Although T710 did not directly contribute to TM dimerization, it played a structural role via intramolecular H-bonds with the F706 carbonyl group (Figure 7A–C). Residues F718, I715, and L714 from the C-terminal end of either helix were involved in a close-range hydrophobic network. The aromatic rings of F718 were stacked against each other, and the aliphatic side chains of I715 and L714 provided further stability through nonpolar interactions. The S2-TM dimer was further stabilized by an additional interaction involving the L714 side chains from both helices (Figure 7E). However, I719 was not observed to contribute to the packing interaction of the dimer because its side chains were extended towards the hydrophobic core of the bilayer. Furthermore, it is possible that residues M707 and L722 play a key role in dimer formation because M707 remained proximal to F706 and because the L722 side chains exhibited a reciprocal interaction over the course of the simulation (Figure 7D–F and Table 3).

To confirm the helix–helix interaction of the TM domains observed in the full-length TLR3, we simulated the NMR structure of the TLR3-TM dimer in a POPC bilayer for 200 ns (S4). The helix–helix interface of the S4-TM exhibited an identical network of interacting residues as found in the TM dimers of the full-length TLR3, with comparable intermolecular distances. S4-TM residues M707 and L722 also demonstrated consistent hydrophobic contact with F706 and L722, respectively, which was also seen in S2-TM. However, the S4-TM helices displayed a larger crossing angle and greater distance between helical axes during the simulation (Supplementary material, Figure S5A–D). This might be due to the absence of upstream ECD and downstream TIR domains, which would have provided the individual TM helices with increased flexibility in the bilayer (Supplementary material, Figure S5E–G). The hydrophobic mismatch between S4-TM and the bilayer core was clearly evident by a sharp curvature and tilt of subunit B of the TM dimer to overcome energetic penalty. A comparison of BFE between the TM segments of different full-length receptors showed that the S2-TM dimer had a greater binding affinity (BFE = −118.7 ± 22.32 kJ mol^−1^) than the other TM dimers (Table 4). The binding affinity of S4-TM was ranked second, suggesting that the helix–helix interface of S2-TM led to a reasonably stable full-length TLR3 in the membrane-bound dynamic condition.

### 2.5. S2-TIR Domains Represent the Most Stable Dimer Interface of TLR3

**Figure 8:**
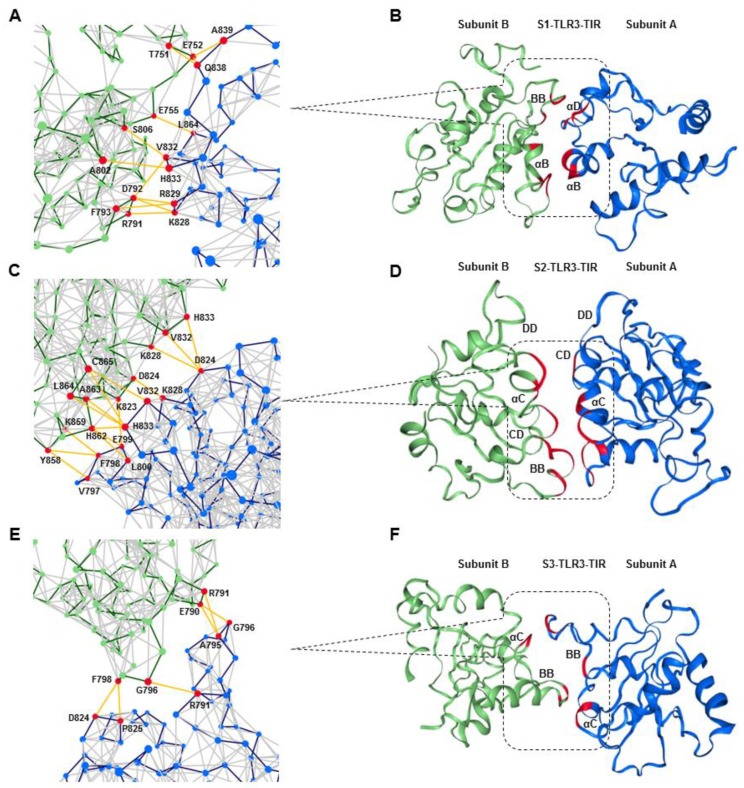
Packing interaction of the TIR domains. (**A**,**C**,**E**) A network view of representative TIR domains extracted from the low-energy ensemble of the molecular dynamics trajectories. Subunits A and B are colored in blue and green, respectively. Each node (represented as a dark circle) of the network represents a Cα atom. The dark-blue or dark-green edges represent backbone contact, while the light-grey edges indicate nonbonded interactions. The orange lines represent inter-residue communication between nodes within a 7 Å distance threshold. (**B**,**D**,**F**) The domain view of the TIR dimers in line with the protein structure network.

Due to the extensive structural transitions of the ECD and TM segments in the phospholipid bilayer, the TIR domains demonstrated distinct molecular topologies and packing interactions during the MD simulations. The TIR–TIR interfaces were analyzed by constructing protein structure networks (PSNs) [34] based on the nonbonded contacts between amino acid residues of the representative low-energy conformations from the MD trajectories (Figure 8). The analysis of inter-residue communication in the PSNs revealed a larger number of nodes (represented by Cα atoms) with high “betweenness” values in the S1- and S2-TIR dimer interfaces compared to the S3-TIR interface. Experiments have shown that residues with statistically significant high betweenness values are often correlated with the hotspot residues of a given protein–protein interface [35]. Most of the nodes in S1- and S2-TIR PSN were interconnected by multiple edges representing interactions within a 7 Å cutoff distance (Figure 8A–D). On the other hand, the S3-TIR dimer interface exhibited poorly connected nodes, indicating a possible dimer dissociation during MD simulations (Figure 8E,F). Specifically, the contact surface of the S2-TIR complex involved a greater number of residues compared to S1-TIR and the local subnetwork spans a relatively broader surface area along the interface, comprising αC helix and CD, DD, and BB loops of both monomers (Figure 8D). The possible dimer dissociation in S3-TIR dimer resulted in the exposure of buried surfaces to the solvent (Figure 8E,F).

Analysis of intermolecular H-bonds (Supplementary material, Table S11) and salt bridges (Supplementary material, Table S12) in the starting structure and their average distances along the MD trajectory found that S2-TIR formed a relatively stronger dimer interface compared to others. We observed that structural transitions in the upstream ECD and TM domains resulted in the loss of a few existing H-bonds and salt bridges between TIR monomers followed by the formation of new polar contacts. A comparison of the average buried surface area (BSA) of the TIR domains along the MD trajectories demonstrated that only the S2-TIR dimer had an appropriate BSA [36], which was greater than S1- and S3-TIR (Table 5). Moreover, based on the solvation energy effect (Δ^i^G) on each interfacing residue, S2-TIR exhibited a relatively lower interface energy (i.e., higher affinity) among the monomers as compared to others. Calculation of BFE clarified that only a S2-TIR dimer exhibits a high-affinity interaction among the monomers, while S1- and S3-TIR demonstrate positive energy values, indicating a repulsive behavior between monomers (Table 6). These results indicate that the packing strength of S2-TIR domains plays an important role in the overall stability of the full-length TLR3 during MD simulations.

**Figure 9:**
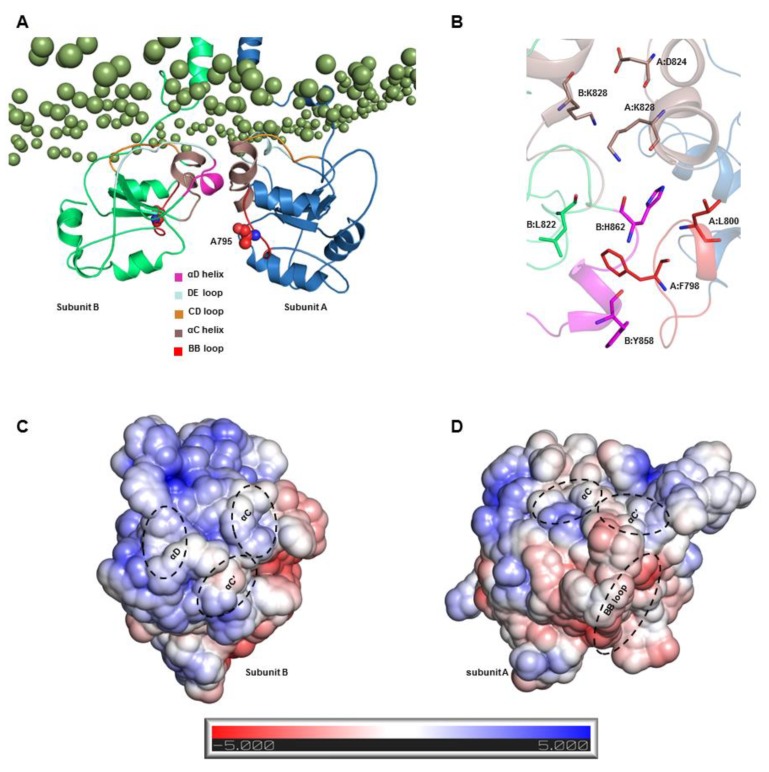
Illustration of the intermolecular interactions and electrostatic properties of the S2-TIR dimerization complex. (**A**) The representative low-energy dimer interface of the S2-TIR domains: The structural elements with significant interactions with either phospholipids or their monomer counterparts are colored differently for clarity. The important BB loop residue, A795, is represented by sphere model in both subunits. (**B**) Zoomed view of the polar and nonpolar interactions occurring at the dimer interface of the S2-TIR domain. (**C**,**D**) The electrostatic potential surface of the two subunits of the S2-TIR dimer: The location of interfacing segments is indicated with dashed circles.

As the S2-TIR dimer exhibited an energetically stable interface area with a greater number of intermolecular contacts, we analyzed its packing interaction based on a representative low-energy TLR3 structure extracted from 173.6-ns MD simulation trajectory. The TIR domains were found to be partially absorbed into the lower leaflet of the bilayer, where the surface-exposed residues from the αD helix and the CD and DE loops facilitated membrane anchoring through numerous electrostatic and van der Waals contacts with the phospholipid headgroups (Figure 9A). The BB loop of subunit A remained solvent-exposed, whereas that of subunit B was mostly buried between the two domains. Residue A795, reported to be essential for the recruitment of TRIF [37], was completely solvent-exposed in subunit A, while it had a BSA of only 5.64 Å² at the dimerization interface in subunit B. The main homodimerization interface involved contact between the αC and αD helices of subunit B and the αC helix and the BB loop of subunit A (Figure 9A). The αC helix from both subunits faced one another, while the αD helix of subunit B was packed against the BB loop of subunit A. In the crystal structures, TLR2-TIR and TLR6-TIR domains were found to be stabilized by disulfide linkages [26,27]. However, in TLR3-TIR, there is a glutamate (E755) at the corresponding position of C640 in TLR2-TIR (Supplementary material, Figure S6) but the cysteine (C827) homologous to C712 in TLR6-TIR was conserved. Although there was no clear disulfide linkage between two monomers in the starting model, the optimized S2-TIR dimer exhibited a close contact between C827 (αC helix) of both monomers. Nevertheless, based on the cytosolic localization of TLR3-TIR domain, C827 in both monomers are unlikely to form a disulfide bond because this has been found to be unusual for proteins under the reducing condition of the cytoplasm where cysteine residues are frequently modified by S-glutathionylation reaction [38,39]. The residues F798 and L800 (BB loop) of subunit A formed hydrophobic contacts with residues Y858 and H862 (αD helix) of subunit B (Figure 9B), while residues K828 and D824 (αC helix) of subunit A make contacts with residues L822 and K828 (αC helix) of subunit B through electrostatic interactions at the dimer interface. These residues were found to bury a large portion of their surface areas by the dimer formation (Supplementary material, Table S13). The electrostatic potential surface around each subunit revealed that the main dimer interface contains both hydrophilic and nonpolar charges that drive the packing interaction between the TIR dimers (Figure 9C).

## 3. Discussion

TLR activation consists of a complex assembly of multimeric receptor and adaptor molecules in the cell or endosomal membrane [40]. Of these, ligand-induced homo/heterodimeric receptors are considered the basic signaling unit required for signal transduction [9]. Here, we studied three membrane-solvated models of 2:1 TLR3-dsRNA homodimerization complexes and identified the model most likely to represent the signaling-competent receptor in a physiological environment. The TIR domain of each full-length receptor was constructed via homology modeling using the crystal structures of either the TLR2-, TLR6-, or TLR10-TIR homodimer as templates. A comparative analysis of three MD trajectories suggested that the TLR3-TIR homodimer built from the TLR6-TIR domain led to a full-length receptor with the stability necessary to maintain key intermolecular interactions with the ligand and with the membrane, as exhibited by the native form.

Our simulation data indicated that the long, flexible juxtamembrane loops of TLR3 allow for the simultaneous bending of the ECD and TIR domains on both surfaces of the membrane. The cytoplasmic linker connecting the TM and TIR domains has been reported to be essential for the subcellular targeting of TLR3 toward the endosomal membrane [41]. The juxtamembrane regions are also believed to play an important role in the membrane-anchoring mechanisms of TLR4 [29,42,43,44]. A previous study of the chimeric TLR3-TLR9 receptor has demonstrated that TLR3 can also be localized to the plasma membrane by means of its ECD but that endocytosis of the receptor-ligand complex and endosomal acidification are required for efficient signaling [45]. We observed that the membrane-anchored TLR3 progressively tilts on the bilayer surface due to the electrostatic attraction between the charged microdomains of both the protein and phospholipids. Although the ECD exhibited a sharp tilt on the membrane, the LRR-NT was partially absorbed by the lipid headgroups; in contrast, the LRR-NT of our previously reported TLR4 model was completely buried in the bilayer surface [29]. It is possible that the negatively charged dsRNA that spans the entire length of the TLR3-ECD restricted the insertion of LRR-NT into the membrane surface, owing to the electrostatic repulsion of the phospholipid headgroups. The role of LRR-NT in the tilting and membrane anchoring of TLR3 seems to agree with the lateral clustering model of TLR3, where multiple ECDs cluster on their lateral surfaces to recognize dsRNA molecules that are up to 90 base pairs long [46]. The convex surface spanning LRRs 3–7, which is distinct from the N-terminal dsRNA-binding site (i.e., LRR-NT and LRRs 1–3), mediates the lateral clustering of ECDs. This indicates that tilted ECDs may facilitate the lateral clustering of TLR3 even though LRR-NT is partially buried in the membrane during ligand-induced signal transduction.

The bound dsRNA retained its structural integrity through the stable interaction with TLR3, indicating that the dynamic behavior of the ECD does not significantly alter the dsRNA binding pattern. The X-ray crystal structure of mouse TLR3-ECD (PDB ID: 3CIY) demonstrated that dsRNA forms two major contacts with the glycan-free surfaces of the ECD: one at the C-terminal site (site I) and the other at the N-terminal site (site II). Site I comprises the conserved residues N515, N517, H539, N541, and R544, while site II consists of residues H39, H60, R64, F84, T86, H108, and E110. The mutations H539E and N541A at site I and the mutations H39A and H60A at site II abrogated the TLR3 response towards dsRNA, indicating these residues are important for ligand recognition [10]. The most critical electrostatic interaction occurs between the imidazole rings of histidine residues and the phosphate groups of the dsRNA backbone [9]. The S2-TLR3 model exhibited a stable interaction between H39/H60 and the dsRNA backbone over the course of the simulation. Although the site II residues of S2-TLR3 formed several transient H-bonds and salt bridges with the phosphate groups of dsRNA, the electrostatic and van der Waals forces were crucial for the stronger binding affinity of the receptor–ligand complex. The variable H-bond/salt bridges at site II may be due to the progressive tilt of the ECD and the slightly twisted dsRNA backbone during the simulation. Previous studies have shown that dsRNA binds to a preformed TLR3 homodimer stabilized by interactions between the LRR-CT domains and that an intact dimerization site is required for both ligand binding and downstream signaling [47,48]. The homodimerization site consists of two H-bond pairs, D648/T679 and G652/H682, and the structural proline P680. Mutational analysis revealed that D648A, T679A, and P680L variants are inactive against dsRNA whereas H682A and E652A are partially active [33]. Our analysis revealed that S2-TLR3 displays an intact homodimerization interface supported by stable spatial contacts among D648/T679 and G652/H682 residue pairs. This result suggests that the equilibrated model of full-length S2-TLR3 embedded in the phospholipid bilayer might represent the physiologically relevant state of an activated receptor.

The tilted architecture of the ECD is followed by the tilting and curvature of the TM helices, causing severe topological changes in the TIR domains. The minor distortion of TM helices is a regular phenomenon that occurs due to a hydrophobic mismatch with the bilayer core [43]. However, the helical transformation of TLR3 was largely distinct from the TLR4-TM previously reported [29,42]. This indicates that the orientation and conformational changes of each TLR type may vary, depending on their location in the membrane or the lipid composition of that particular area. Membrane proteins usually exist within specialized microdomains called lipid rafts, which contain different arrangements of localized lipids [49]. Therefore, the liquid order of a particular membrane microdomain might have an impact on the orientation behavior of membrane proteins, as shown here [50]. It has been reported that the NMR structure of an isolated TLR3-TM domain in dodecylphosphocholine micelles exists as a right-handed dimer with a helix-crossing angle of –51.1° [51]. The substantial tilt and curvature and the consistent crossing angle of TM helices in our simulations indicate that the hydrophobic mismatch shapes the TM domain organization of full-length TLR3 in the cell membrane [52]. As in the full-length structure of TLR4 [29], the membrane-spanning segment of TLR3 is a relatively long helix of ~30 residues, which included 21 residues of the TM domain and rest from the juxtamembrane segments. Analysis of the amino acid composition of the TM domains revealed the presence of several aromatic residues and relatively polar residues. We observed a cluster of aromatic and aliphatic residues that were entangled in the aromatic stacking and hydrophobic contacts at the C-terminal end of the TM bundle. Partial charges on aromatic side chains can also interact with other aromatic or polar side chains through electrostatic interaction [53]. We also found that residues M707 and I722 from either end of the TM helix could contribute to the dimerization surface through their long, branched aliphatic side chains. The isolated TLR3-TM domains have the ability to form homodimers or trimers, stabilized by aromatic stacking and van der Waals interaction between the bulky side chains [11]. While both the dimeric and trimeric forms of TM domain are possible in a micellar environment, the oligomer form of the entire TLR3 molecule in a biological membrane (such as endosomal or lysosomal membranes) needs further experimental clarification.

Ligand recognition enables conformational changes in the TLR structure, thus bringing the TIR domains into close proximity [54]. Because the experimental structure of the TLR3-TIR domain is unknown, computational models have been used to understand the structure, dynamics, and molecular basis of TLR3-mediated signal transduction [55,56]. Based on the sequence homology, the crystal structure of the TLR10-TIR domain seems to be the most suitable template for modeling TLR3-TIR structure (Supplementary material, Figure S6). Although the prediction of dimeric TLR3-TIR architecture is challenging, especially in the absence of biochemical or mutagenesis data, we used multiple homology models followed by MD optimizations to address such problem. Our results showed that the TIR domain architecture of TLR2 or TLR10 was not compatible with the full-length TLR3 in the phospholipid bilayer because the interaction between monomers under dynamic condition was inconsistent or relatively less stable. On the other hand, the architecture of the TLR6-TIR dimer was remarkably stable with an appropriate packing interaction between monomers in the dynamic condition. The main dimerization interface in the TLR6-TIR crystal structure is defined by reciprocal contact between the residues from the CD loop, DD loop, and αC helix of both monomers [27]. Our equilibrated S2-TLR3 structure exhibited a TIR-TIR interface involving residues from the αC and αD helix and the CD and DE loops of both monomers. The BB loop of one subunit was completely solvent-exposed, while that of the other was partially involved in dimer packing. The BB loop of several TLRs contains the conserved RDXXP motif, which is critical for receptor oligomerization and adaptor recruitment [54]. In TLR3, the proline of the RDXXP is replaced by an alanine (A795), which has been found to be essential for the recruitment of the TRIF adaptor [37]. Our analysis of the S2 MD trajectory revealed that A795 is excluded from the homodimerization interface over the course of the entire simulation, suggesting that the BB loop together with A795 can be readily available for TRIF binding. Thus, we assume that the S2-TLR3 model better explains the signaling-competent form of the full-length receptor bound to an agonist. Previous reports have demonstrated that the sequential phosphorylation of Y858 and Y759 is required to initiate TLR3 signaling for the activation of IRF3 and NF-κB [57,58,59]. We found that the side chain of Y858 (αD helix) face opposite to F798 (BB loop) and its hydroxyl group is exposed to the solvent. Likewise, the hydroxyl group of Y759 (βA strand) faced the surface, increasing the likelihood of a phosphorylation reaction. This suggests that the architecture of the S2-TIR domain in full-length TLR3 agrees with current biochemical and mutagenesis data regarding TLR3 signal transduction.

In conclusion, we modeled the structural organization of full-length TLR3 and identified key molecular interactions that are critical for receptor activation under physiological conditions. The ECD of TLR3 is able to orient towards either direction of the membrane surface, while TM helices tilt with a consistent crossing angle to overcome the hydrophobic mismatch. The upper surfaces of the TIR domain were partially absorbed into the lower surface of the membrane bilayer, while the dimer interface comprises the αC, αD helix, and the CD and DE loops from both monomers in an equilibrated TLR3 receptor. The BB loop of one subunit contributes to the dimer packing, while the other subunit remained solvent-exposed throughout the MD simulation, confirming the importance of this segment in adaptor recruitment by the activated receptor (Supplementary Materials, Model S1). Collectively, our MD simulation data provides crucial insights into the signaling-competent form of the full-length TLR3-dsRNA complex that could be used to inform the future design of therapeutic peptide or small molecule drug candidates.

## 4. Materials and Methods

### 4.1. Construction of the Full-Length TLR3 Models

The full-length structure of TLR3 (residues 24–904) was modeled in successive stages as follows. First, the mouse TLR3-ECD-dsRNA complex was obtained from the PDB (PDB ID: 3CIY [10]). The human form of TLR3 (UniProt ID: O15455) was modeled via homology modeling using 3CIY as the template on the SWISS-MODEL server [60]. Second, the NMR structure of the human TLR3-TM domain in its dimeric form was obtained from the PDB (PDB ID: 2MK9) [11]. Third, three separate models of the TIR domain were constructed using homology modeling on the SWISS MODEL server with TLR2-TIR (PDB ID: 1O77) [26], TLR6-TIR (PDB ID: 4OM7) [27], and TLR10-TIR (PDB ID: 2J67) [28] employed as homologous templates. A BLAST search against the PDB revealed that the TLR3-TIR domain has sequence identities comparable to the TLR2-, TLR6-, and TLR10-TIR domains. However, these TIR domains have different dimer interfaces and distinct adaptor-binding surfaces. Therefore, we modeled three separate structures for the TLR3-TIR domain with different homodimerization interfaces. The monomeric TLR3-TIR domains were superposed onto the dimeric templates to obtain the dimeric TLR3-TIR domains. Fourth, the ECD, TM, and TIR domains of TLR3 were aligned on a straight axis using Discovery Studio Visualizer (DSV 4.0; Dassault Systèmes, San Diego, USA) so that the C-terminus and the N-terminus of each domain faced each other within a covalent-bonding distance. This structural organization was subsequently utilized as the template for building the full-length structure of TLR3 on the SWISS-MODEL server. The inter-domain loops were further optimized using the MODLOOP server [61]. Ultimately, we obtained three full-length TLR3 models with the homodimerization interfaces of the TLR2-, TLR6-, or TLR10-TIR domain.

### 4.2. Construction of the Lipid Bilayers and the Packing of Lipids around the Modeled TLR3

TLR3 models were simulated in separate POPC bilayers with a different number of phospholipids. Initially, a pre-equilibrated bilayer of 128 POPC lipids was obtained from the Peter Tieleman website (http://www.ucalgary.ca/tieleman). The bilayer was then replicated in the X and Y directions using the *gmx conf* tool in the GROMACS 5.1.5 [62] simulation package so that the TLR3-ECD could be accommodated in lateral directions. The bilayer was further optimized using a round of energy minimization and MD simulation (100 ns). The TM domains of full-length TLR3 were manually aligned, matching the hydrophobic segment of the bilayer, and the lipids were packed around the protein using the InflateGRO methodology [63].

### 4.3. MD Simulations of the TLR3-dsRNA Complexes

All simulations were carried out using a hybrid force field containing AMBER99SB-ILDN parameters for protein and Berger-lipid parameters for lipid atoms [64]. All histidine amino acids on the ECD of TLR3 were protonated (i.e., H on both ND1 and NE2 atoms) using the interactive histidine (-his) flag of GROMACS to mimic their protonation status inside the endosomal compartment (i.e., pH ≤ 6.5). Energy minimization and the MD simulations were conducted using GROMACS. The simulation systems were solvated with TIP3P water molecules and neutralized by adding an appropriate amount of counterions (Na^+^/Cl^−^). Energy minimization was conducted using the steepest descent algorithm until the maximum force (F_max_) of 1000 kJ mol^−1^ nm^−1^ had been reached. Temperature equilibration was carried out using an NVT ensemble at 271 K via the V-rescale method, and the pressure was equilibrated using an NPT ensemble at 1 bar with the Parinello–Rahman algorithm. During temperature and pressure equilibrations, the positions of the heavy backbone atoms were harmonically restrained. The production run was carried out using an NPT ensemble without backbone restraints for 200 ns. Each TLR3-dsRNA system was simulated three times by assigning random velocity during the NVT equilibration. Long-range electrostatic interactions were calculated by the particle mesh Ewald method, while the short-range electrostatic and van der Waals interactions were calculated by specifying a 12-Å cutoff distance. Periodic boundary conditions were applied to all simulations, and bonds involving hydrogen atoms were constrained using the linear-constraint-solving algorithm. Trajectory data were saved at time intervals of 2 ps. Data analysis and visualization were conducted using visual molecular dynamics (VMD) [65], DSV, PyMOL (Schrödinger, LLC, New York, NY, USA), Grace (http://plasma-gate.weizmann.ac.il/Grace/), and other built-in tools in GROMACS.

### 4.4. Electrostatic Potential Surface

The electrostatic potential surfaces were modeled using the *apbsplugin* tool (https://pymolwiki.org/index.php/Apbsplugin) in PyMOL. The solvent-accessible surface area (SASA) of the input structures was calculated by solving the linearized Poisson–Boltzmann (PB) equation with a bulk solvent radius of 1.4 Å and a dielectric constant of 78. The electrostatic isosurfaces (positive and negative surfaces) were viewed using a contour (kT/e) value of 1.

### 4.5. Free Energy Landscape (FEL)

The FEL was generated to identify representative low-energy conformations of the TLR3 model. The calculation was performed using the GROMACS *gmx sham* tool, and the landscape was plotted using Mathematica software (Version 11.2; Wolfram Research, Inc., Champaign, IL, USA). The input trajectories for FEL calculations were prepared by writing all conformations of the largest cluster in the whole MD trajectories using *gromos* algorithm.

### 4.6. Model Validation

The stereochemical parameters of the starting TLR3 models were evaluated in the Structure Analysis and Verification Server (SAVeS) with the Verify 3D [66] and ERRAT [67] programs. The models were further validated using the Protein Structure Analysis (ProSA)-Web [68] and Rampage servers [69] before carrying out MD simulations.

### 4.7. Binding Free Energy (BFE)

The BFE of the TLR3–dsRNA complexes was calculated using the molecular mechanics/Poisson–Boltzmann surface area (MM/PBSA) method [70]. The calculation was conducted using the g_mmpbsa program [71] with Equation (1):

ΔG_bind_ = (G_complex_) − (G_protein_) − (G_ligand_) (1)

where G_bind_ is the total BFE and where G_complex_, G_protein_, and G_ligand_ are the average free energies of the complex, the protein, and the ligand, respectively. The free energy of each component was calculated using Equation (2):

G = G_bond_ + G_ele_ + G_vdw_ + G_pol_ + G_npol_ − TS (2)

where G_bond_ is the sum of the bond, angle, and dihedral energies and G_ele_ and G_vdW_ are the electrostatic and van der Waals energies, respectively, derived from the calculation of the molecular mechanics energy. G_pol_ and G_npol_ are the polar and nonpolar contribution to the solvation energy, respectively, with G_pol_ obtained by solving the PB equation and G_npol_ estimated from its linear relationship with the SASA. The configurational entropy (TS) is typically ignored because of the greater computational costs and overestimated BFE values [72].

### 4.8. Protein Structure Network

The Network Analysis of Protein Structure (NAPS) server [73] was used to generate unweighted residue interaction network diagrams based on noncovalent interactions between amino acid residues. The Cα atoms were chosen as nodes and interactions between nodes were identified using a distance threshold of 7 Å and a residue separation value of 1.

### 4.9. Miscellaneous Properties

The BSA at interfaces of different TIR dimers and interaction energies were calculated using the *gmx sasa* tool of GROMACS. Analysis of dsRNA properties was carried out using 3DNA software [74]. The physiochemical properties of the protein domains were analyzed with the ExPASy–ProtParam tool [75]. All the helical properties were analyzed using *gmx bundle*, *gmx helix*, or *gmx helixorient* tools of GROMACS.

## Figures and Tables

**Table 1:** The decomposition of the binding free energies (kJ mol^−1^) of TLR3-dsRNA complexes.

	^1^ Δ_vdW_	^2^ Δ_elec_	^3^ Δ_ps_	^4^ Δ_SASA_	^5^ ΔG_Total_
**Subunit A**					
S1-TLR3-dsRNA	−62.13 ± 17.88	−5754.51 ± 128.30	1267.30 ± 120.76	−10.79 ± 1.91	−4560.13 ± 65.34
S2-TLR3-dsRNA	−65.09 ± 11.99	−5744.62 ± 124.09	1170.13 ± 110.45	−11.68 ± 4.13	−4651.26 ± 68.97
S3-TLR3-dsRNA	−55.04 ± 13.05	−5236.47 ± 125.15	1256.26 ± 129.08	−18.23 ± 2.03	−4053.48 ± 66.08
**Subunit B**					
S1-TLR3-dsRNA	−97.99 ± 14.01	−5474.26 ± 104.82	1811.79 ± 37.17	−17.72 ± 16.45	−3778.18 ± 62.93
S2-TLR3-dsRNA	−92.51 ± 18.44	−5394.85 ± 111.91	1863.41 ± 47.51	−14.43 ± 16.75	−3638.38 ± 65.44
S3-TLR3-dsRNA	−94.09 ± 19.72	−5264.13 ± 119.56	1972.11 ± 75.21	−17.17 ± 16.73	−3403.28 ± 69.40

^1^ Van der Waals energy; ^2^ electrostatic energy; ^3^ polar solvation energy; ^4^ solvent accessible surface area energy; ^5^ total binding free energy; TLR3, Toll-like receptor 3; dsRNA, double-stranded RNA. S1, S2, and S3 represent sets 1, 2, and 3, respectively.

**Table 2:** Average distance between histidine imidazole and dsRNA phosphate groups.

	TLR3			dsRNA		TLR3			dsRNA	
	Subunit	Residue		Subunit	Residue	Subunit	Residue		Subunit	Residue
			* Dist. (Å)					Dist. (Å)		
S1-TLR3-ECD										
	A	H539	3.93 ± 1.17	D	U22	B	H539	6.69 ± 0.48	C	G23
	A	H539	4.02 ± 0.53	D	C23	B	H539	3.5 ± 0.27	C	A22
	A	H39	7.55 ± 1.55	C	C7	B	H39	10.0 ± 4.59	D	C8
	A	H60	3.71 ± 0.34	C	C7	B	H39	10.7 ± 2.93	D	G7
	A	H108	3.74 ± 0.35	C	U5	B	H60	6.9 ± 3.76	D	G7
						B	H108	4.46 ± 1.35	D	C6
S2-TLR3-ECD										
	A	H539	8.13 ± 0.42	D	C23	B	H539	4.12 ± 0.33	C	A22
	A	H39	2.79 ± 0.1	C	C7	B	H539	4.64 ± 0.78	C	A21
	A	H39	3.66 ± 1.2	C	G6	B	H39	3.83 ± 1.19	D	C8
	A	H60	6.83 ± 0.73	C	G6	B	H60	3.48 ± 0.24	D	G7
	A	H108	3.8 ± 0.47	C	U5	B	H108	3.6 ± 0.25	D	C6
S3-TLR3-ECD										
	A	H539	7.03 ± 0.57	D	C24	B	H539	11.2 ± 1.44	C	G23
	A	H539	3.47 ± 0.26	D	C23	B	H539	5.75 ± 1.36	C	A22
	A	H39	12.73 ± 1.45	C	C7	B	H39	14.14 ± 2.36	C	C34
	A	H60	3.78 ± 1.1	C	G6	B	H39	12.68 ± 2.19	C	A33
	A	H108	3.89 ± 0.36	C	U5	B	H60	12.71 ± 2.27	C	C34
						B	H108	12.03 ± 2.31	C	A35

* Distance between the center of mass of the histidine imidazole rings and dsRNA phosphate groups averaged over all structural frames in the molecular dynamics trajectories; TLR3, Toll-like receptor 3; S1, S2, and S3 represent sets 1, 2, and 3, respectively.

**Table 3:** Distance between the center of mass of important TM domain residues in three representative simulations.

	Subunit	Residue	* Dist. (Å)	Subunit	Residue
S1-TLR3-TM					
	A	F718	3.96 ± 0.41	B	I715
	A	I715	3.74 ± 0.31	B	F718
	A	F706	7.4 ± 1.19	B	F706
	A	M707	7.57 ± 2.71	B	F706
	A	F706	4.30 ± 0.91	B	M707
	A	L722	3.77 ± 0.60	B	L722
S2-TLR3-TM					
	A	F718	5.21 ± 1.03	B	F718
	A	L714	7.61 ± 1.07	B	I715
	A	L714	4.39 ± 1.15	B	L714
	A	F706	7.80 ± 1.79	B	F706
	A	M707	3.33 ± 0.56	B	F706
	A	F706	5.15 ± 1.93	B	M707
	A	L722	3.22 ± 0.32	B	L722
S3-TLR3-TM					
	A	F718	4.03 ± 0.48	B	I715
	A	I715	7.09 ± 0.59	B	L714
	A	F706	5.02 ± 1.68	B	F706
	A	M707	7.11 ± 1.56	B	F706
	A	F706	11.81 ± 1.25	B	M707
	A	L722	8.68 ± 1.02	B	L722

* Distance between the center of mass of the side chain atoms averaged over all structural frames in the molecular dynamics trajectories; S1, S2, and S3 represent sets 1, 2, and 3, respectively.

**Table 4:** Decomposition of the binding free energy (kJ mol^−1^) between TM domains of TLR3.

System	^1^ Δ_vdW_	^2^ Δ_elec_	^3^ Δ_ps_	^4^ Δ_SASA_	^5^ ΔG_Total_
S1-TM	−115.49 ± 17.31	9.24 ± 2.85	46.47 ± 10.1	−15.55 ± 2.23	−75.33 ± 12.85
S2-TM	−183.38 ± 21.29	11.74 ± 4.34	75.14 ± 12.3	−22.2 ± 1.8	−118.7 ± 22.32
S3-TM	−132.91 ± 15.77	7.31 ± 2.5	55.29 ± 16.25	−17.62 ± 1.72	−87.93 ± 18.4
^6^ S4-TM	−147.03 ± 15.38	9.29 ± 4.11	59.81 ± 15.56	−21.87 ± 2.3	−99.8 ± 15.02

^1^ Van der Waals energy; ^2^ electrostatic energy; ^3^ polar solvation energy; ^4^ solvent accessible surface area energy; ^5^ total binding free energy; ^6^ NMR structure of an isolated TLR3-TM homodimer. S1, S2, and S3 represent sets 1, 2, and 3, respectively.

**Table 5:** Buried surface area (BSA) and interface energy (ΔiG) of TIR domains in the full-length TLR3.

Domain	BSA (Å²)	Energy (kcal mol^−1^)
S1-TLR3-TIR	722.22 ± 15.23	−3.48 ± 0.29
S2- TLR3-TIR	885.56 ± 14.2	−5.77 ± 0.25
S3- TLR3-TIR	480.45 ± 16.12	−2.64 ± 0.63

Values were averaged over all structural frames in the molecular dynamics trajectories. S1, S2, and S3 represent simulation sets 1, 2, and 3, respectively.

**Table 6:** Decomposition of the binding free energy (kJ mol^−1^) between TIR domains.

System	^1^ Δ_vdW_	^2^ Δ_elec_	^3^ Δ_ps_	^4^ Δ_SASA_	^5^ ΔG_Total_
S1-TLR3-TIR	−289.06 ± 12.06	−459.99 ± 19.11	958.13 ± 12.14	−38.94 ± 3.72	170.44 ± 14.24
S2-TLR3-TIR	−223.41 ± 19.12	−509.07 ± 19.38	643.17 ± 18.59	−31.00 ± 5.29	−120.31 ± 17.44
S3-TLR3-TIR	−155.66 ± 12.29	−123.57 ± 19.45	478.11 ± 21.03	−20.56 ± 4.34	178.32 ± 17.35

^1^ Van der Waals energy; ^2^ electrostatic energy; ^3^ polar solvation energy; ^4^ solvent accessible surface area energy; ^5^ total binding free energy; S1, S2, and S3 represent sets 1, 2, and 3, respectively.

## Supplementary Materials

Supplementary Materials can be found at https://www.mdpi.com/1422-0067/21/8/2857/s1.
