# Supplementary material for: A Computational Probe into the Structure and Dynamics of the Full-Length Toll-Like Receptor 3 in a Phospholipid Bilayer
Source: Int J Mol Sci. 2020 Apr 19;21(8):2857. doi: 10.3390/ijms21082857 (PMC7215789; doi:10.3390/ijms21082857)
Supplement: Supplementary file 1 [file ijms-21-02857-s001.zip › ijms-760659-supplementary/IJMS_Supple Info.pdf]

<Supplementary Materials>

A computational probe into the structure and dynamics of the full-length Toll-like receptor 3 in a phospholipid bilayer

Mahesh Chandra Patra, Maria Batool, Muhammad Haseeb, and Sangdun Choi*

Department of Molecular Science and Technology, Ajou University, Suwon, 16499, Korea

*Corresponding author: sangdunchoi@ajou.ac.kr

The supplementary materials include the following items:

Figure S1. Structural comparison between average (minimized) coordinates from the three replicates of S1-, S2-, and S3-TLR3 simulation sets. (a and d) S1-TLR3. (b and e) S2-TLR3. (c and f) S3-TLR3. (g) Comparison of root mean square deviation between the average-coordinates from the molecular dynamics trajectories.

Figure S2. Analysis of different properties of dsRNA base pair and backbone. (A-F) Base pair parameters of dsRNA in three representative MD simulation trajectories. (G-I) Comparative RMSD curves of dsRNA in all three replicates of the MD simulations.

Figure S3. RMSD parameters of the individual domains (ECD, TM, and TIR) of TLR3 in all three replicates of the MD simulation.

Figure S4. Averaged stability parameters of the phospholipid bilayer during molecular dynamics simulations. (A-C) The density profile of different membrane components. (D-F) The order parameters of the acyl chains of phospholipids. (G-I) The lateral diffusion of lipids in terms of the mean square displacement of phosphate (P8) atoms. Data shown represent the replicate 2 of the MD simulation.

Figure S5. MD simulation of an NMR structure of isolated TM domain of TLR3. (**A**) Helical length. (B) Axial tilt. (C) Distance between axes centers. (D) Helix crossing angle. (E) Membrane solvated model of TM domains at 0-ns. (F) Membrane solvated model of TM domains at 200-ns for comparison. (G) Packing interaction of a representative TM bundle (189.1 ns) extracted from the low-energy ensemble of the Gibbs free energy landscape.

Figure S6. Sequence alignment between the TLR3-TIR domain and the homologous templates. (A) Alignment between the TLR2- and TLR3-TIR domains. (B) Alignment between the TLR6- and TLR3-TIR domains. (C) Alignment between the TLR10- and TLR3-TIR domains. The residues involved in the packing interaction of the dimer are colored red. Residues forming disulfide bonds are colored green. The names of the structural elements comprising the dimer interface are presented at the top of each alignment. Identical residues are indicated with an asterisk (*), residues with strong similarity are indicated with a colon (:), and residues with weakly similar properties are indicated with periods (.). The corresponding key residues in TLR3 are shown in a

box to the right of each alignment. The alignments were generated using Clustal-Omega with default parameters.

Table S1. The top-scoring templates used for the homology modeling of TLR3-TIR domains.

Table S2. Stereochemical parameters of TLR3 models.

Table S3. The molecular composition of the simulation systems in the present study.

Table S4. Calculated area per lipid (APL) and thickness of the membrane bilayer in the three molecular dynamics simulation systems.

Table S5. Intermolecular hydrogen bonds (H-bonds) formed between dsRNA and S1-TLR3.

Table S6. Intermolecular hydrogen bonds (H-bonds) formed between dsRNA and S2-TLR3.

Table S7. Intermolecular hydrogen bonds (H-bonds) formed between dsRNA and S3-TLR3.

Table S8. Average base-pair parameters of dsRNA molecule in the three molecular dynamics trajectories.

Table S9. Average distance between dimer forming residues of the extracellular domains of TLR3.

Table S10. Selected helical properties of the TM helices in the three TLR3 models.

Table S11. Comparison of intermolecular hydrogen bonds (H-bonds) formed at the dimerization interface of TIR domains.

Table S12. Intermolecular salt bridges between TIR domain subunits.

Table S13. Buried surface area (BSA) of key residues at the dimer interface of TIR domains.

Model S1. A representative, low-energy membrane-solvated model of dsRNA-bound full-length TLR3 extracted from 173.6-ns MD simulation.

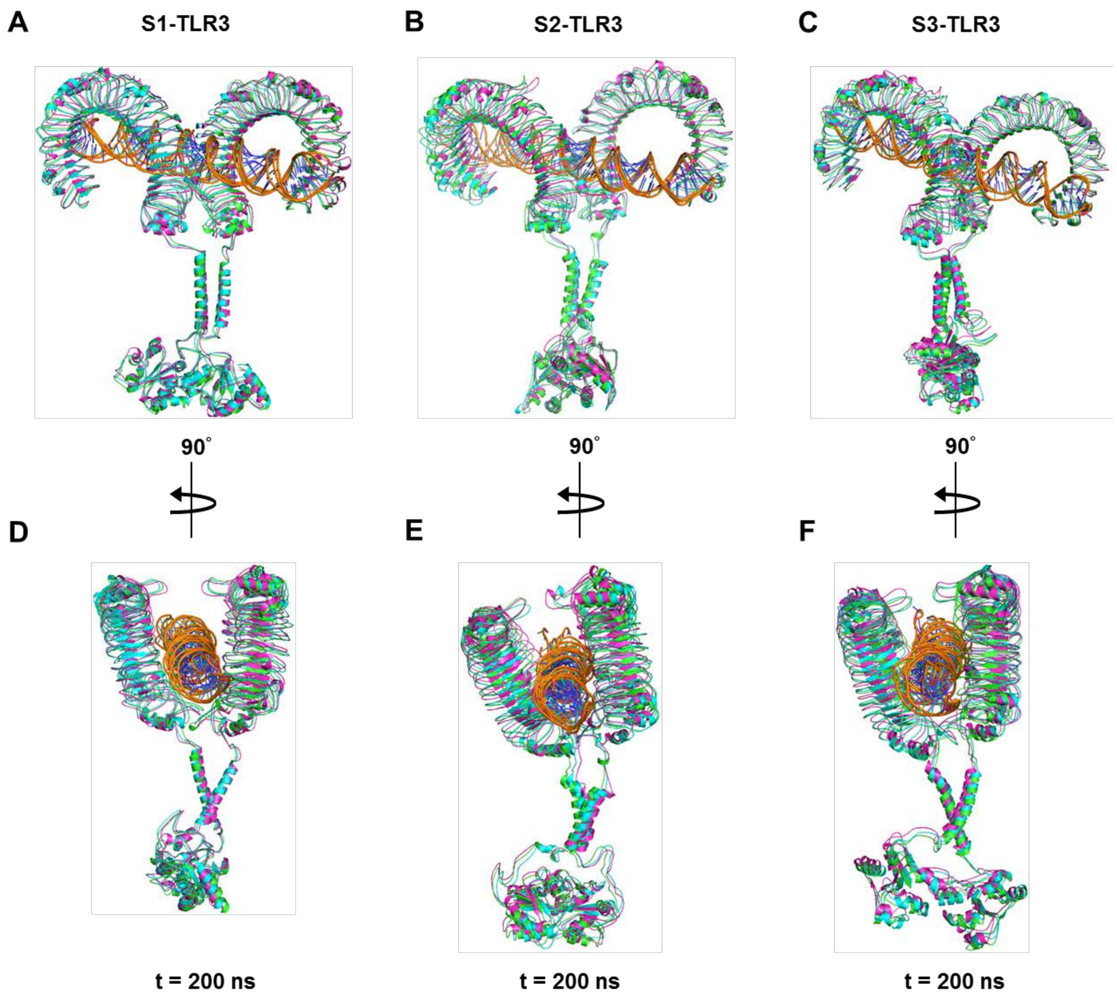

G

RMSD (Å)	S1-TLR3	S2-TLR3	S3-TLR3
Run 1:3	2.1	1.78	3.29
Run 1:2	1.5	1.53	1.91
Run 2:3	2.1	1.71	3.08

Run 1
Run 2
Run 3

Figure S1. Structural comparison between average (minimized) coordinates from the three replicates of S1-, S2-, and S3-TLR3 simulation sets. (A and D) S1-TLR3. (B and C) S2-TLR3. (E and F) S3-TLR3. (G) Comparison of root mean square deviation between the average-coordinates from the molecular dynamics trajectories.

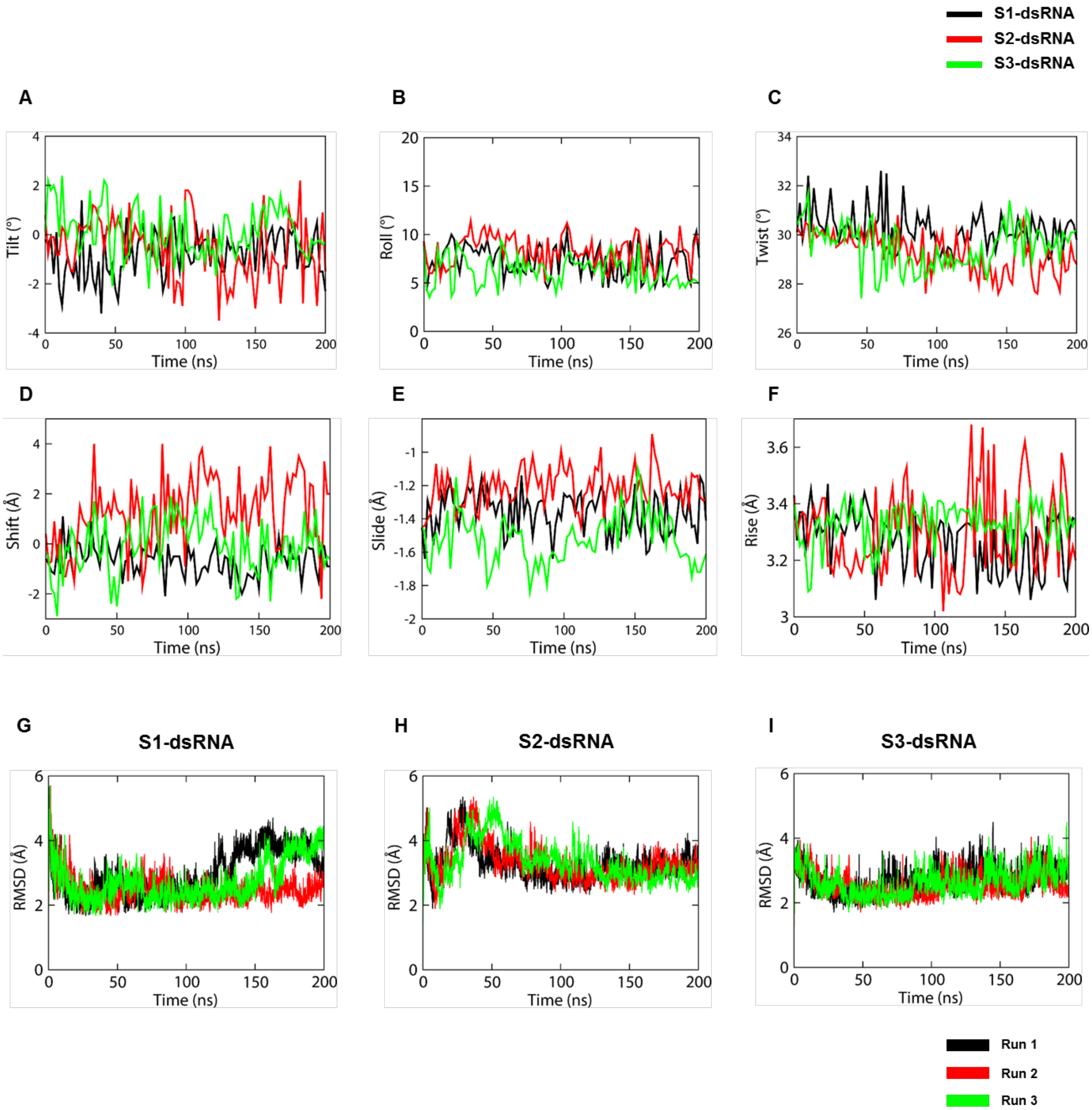

Figure S2. Analysis of different properties of dsRNA base pair and backbone. (A-F) Base pair parameters of dsRNA in three representative MD simulation trajectories. (G-I) Comparative RMSD curves of dsRNA in all three replicates of the MD simulations.

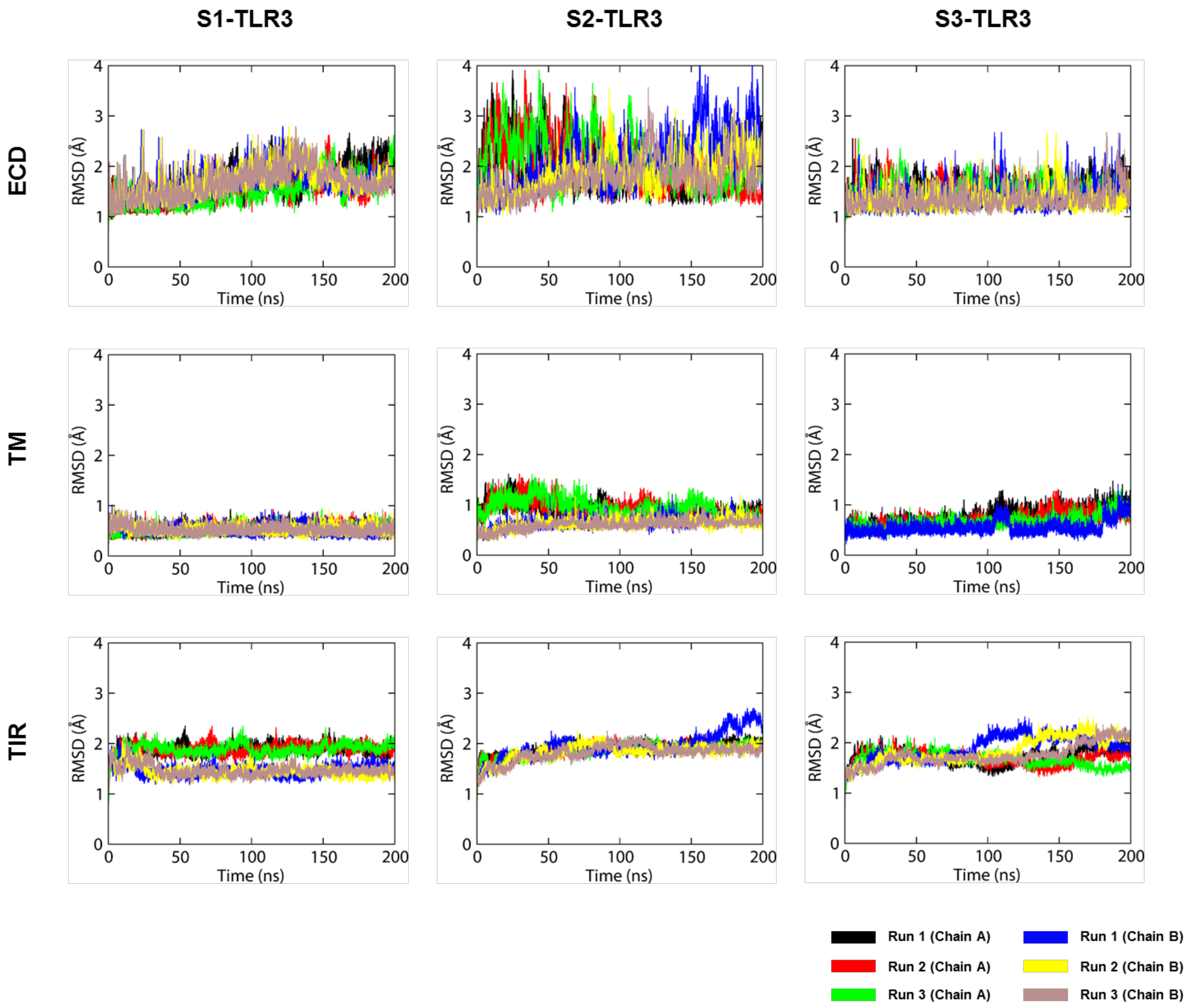

Figure S3. RMSD parameters of the individual domains (ECD, TM, and TIR) of TLR3 in all three replicates of the MD simulation.

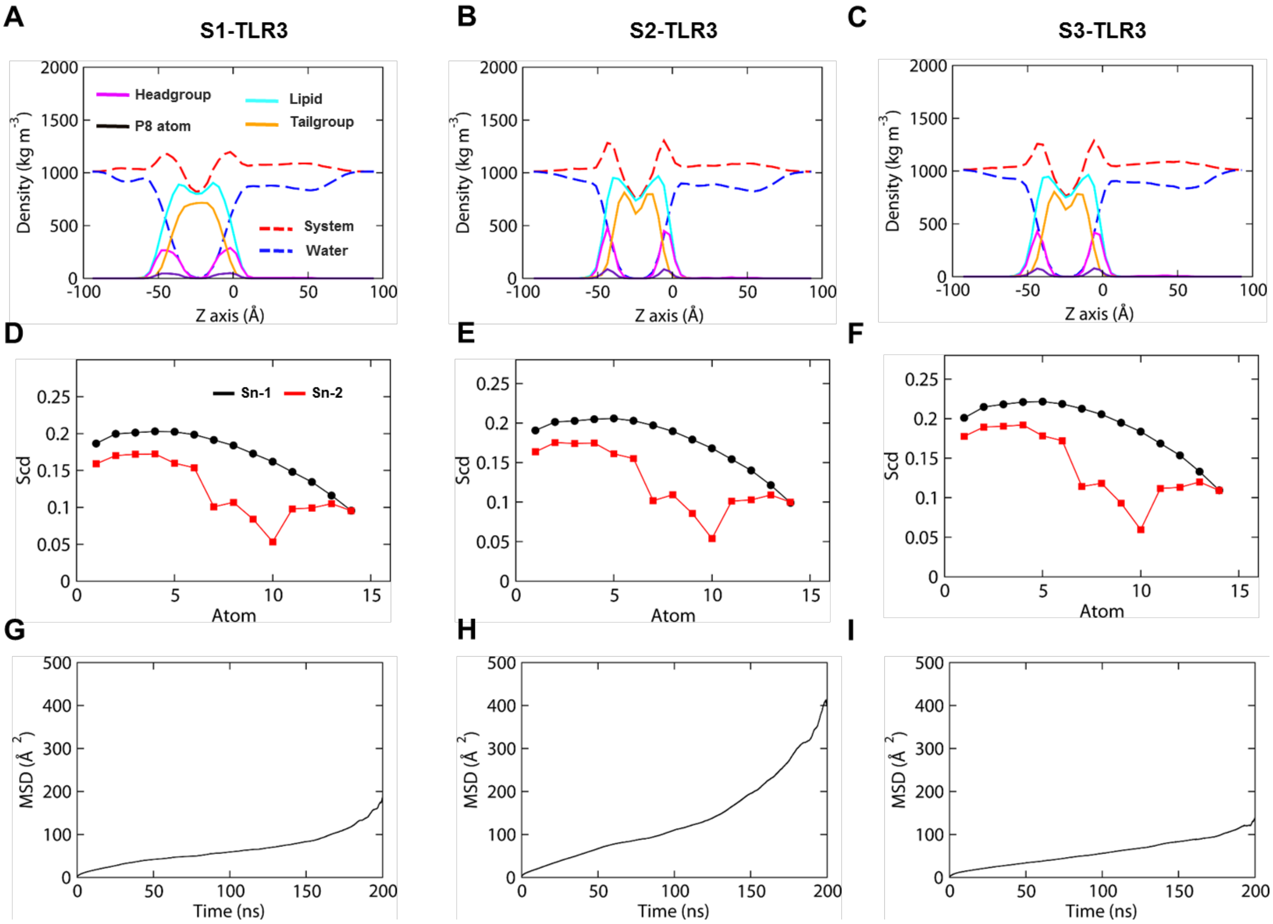

Figure S4. Averaged stability parameters of the phospholipid bilayer during molecular dynamics simulations. (A-C) The density profile of different membrane components. (D-F) The order parameters of the acyl chains of phospholipids. (G-I) The lateral diffusion of lipids in terms of the mean square displacement of phosphate (P8) atoms. Data shown represent the replicate 2 of the MD simulation.

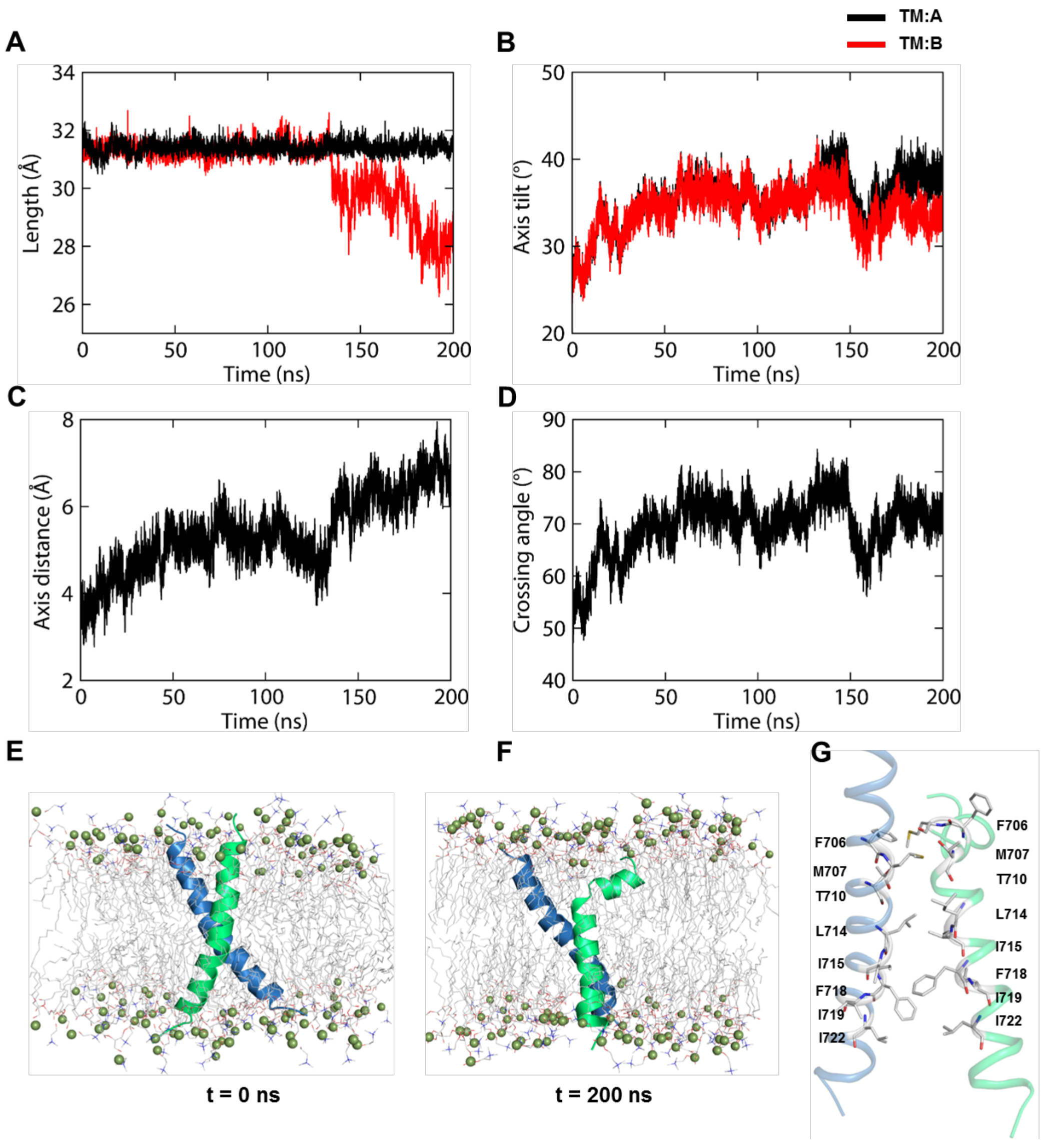

Figure S5. MD simulation of an NMR structure of isolated TM domain of TLR3. (**A**) Helical length. (B) Axial tilt. (C) Distance between axes centers. (D) Helix crossing angle. (E) Membrane solvated model of TM domains at 0-ns. (F) Membrane solvated model of TM domains at 200-ns for comparison. (G) Packing interaction of a representative TM bundle (189.1 ns) extracted from the low-energy ensemble of the Gibbs free energy landscape.

A

```
                     βA                                      βB      BB
TLR2|639-784     ICYDAFVSYSERDAYWVENLMVQELENFNPPFKLCLHKRDFIPGKWIIDNIIDSIEKSHK   60
TLR3|754-896     FEYAAYIIHAYKDKDWVWEH-FSSMEKEDQSLKFCLEERDFEAGVFELEAIVNSIKRSRK   59
                 : * *:: :: :*  ** :  ...:*: :  :*:**.:***  * : :: *::**::*:*
                             αC      αC`                               DD
TLR2|639-784     TVFVLSENFVKSEWCKY-ELDFSHFRLFDENNDAAILILLEPIEKKAIPQRFCKLRKIMN  119
TLR3|754-896     IIFVITHHLLKDPLCKRFKVHHAVQQAIEQNLDSIILVFLEEIPDYKLNHALCLRRGMFK  119
                  :**::.:::*.  **  ::..:  : :::* *: **::** * .  : : :*  * :::

TLR2|639-784     TKTYLEWPMDEAQREGFWVNLRAAIKS   146
TLR3|754-896     SHCILNWPVQKERIGAFRHKLQVA---   143
                 ::  *:**::: :  .*  :*:.*
```

TLR2 -> TLR3
C640 -> E755
Y641 -> Y656
R748 -> A863
C750 -> C865
C673 -> C787
D678 -> D792
F679 -> F793
P681 -> A795
C713 -> C827
L717 -> V832

B

```
                                                                     BB
TLR6|640-784     LQFHAFISYSEHDSAWVKSELVPY-LEKEDIQICLHERNFVPGKSIVENIINCIEKSYKS   59
TLR3|754-896     FEYAAYIIHAYKDKDWVWEHFSSMEKEDQSLKFCLEERDFEAGVFELEAIVNSIKRSRKI   60
                 ::: *:* :: :*. ** ..:     *.:.:::**.**:*  *   :* *:*.*::* *
                         αC      αC`                          DD
TLR6|640-784     IFVLSPNFVQSEWCHY-ELYFAHHNLFHEGSNNLILILLEPIPQNSIPNKYHKLKALMTQ  118
TLR3|754-896     IFVITHHLLKDPLCKRFKVHHAVQQAIEQNLDSIILVFLEEIPDYKLNHALCLRRGMFKS  120
                 ***:: ::::.  *:  :::.* :: :.:. :.:**::** **: .: :     :.::..

TLR6|640-784     RTYLQWPKEKSKRGLFWANIRAAFNMK   145
TLR3|754-896     HCILNWPVQKERIGAFRHKLQVA----   143
                 :  *:** :*.: * *  :::.*
```

TLR6 -> TLR3
I684 -> E789
Q708 -> K823
C712 -> C827
H713 -> K828
L716 -> V832
Y717 -> H833
N746 -> A863
K747 -> H862

C

```
                                                                      BB
TLR10|632-778     VRFHAFISYSEHDSLWVKNELIPNLEKEDGSILICLYESYFDPGKSISENIVSFIEKSYK   60
TLR3|754-896      FEYAAYIIHAYKDKDWVWE-HFSSMEKEDQSLKFCLEERDFEAGVFELEAIVNSIKRSRK   59
                  ..: *:* :: :*. ** :  : .:**** *: :** *  *: *    * **. *::* *
                                   αC                            DD
TLR10|632-778     SIFVLSPNFVQNEWCHY-EFYFAHHNLFHENSDHIILILLEPIPFYCIPTRYHKLKALLE  119
TLR3|754-896      IIFVITHHLLKDPLCKRFKVHHAVQQAIEQNLDSIILVFLEEIPDYKLNHALCLRRGMFK  119
                   ***:: :::::  *:  :.:.* :: :.:* * ***::** ** * :       :.:::

TLR10|632-778     KKAYLEWPKDRRKCGLFWANLRAAINVN 147
TLR3|754-896      SHCILNWPVQKERIGAFRHKLQVA---- 143
                  .:. *:** ::.: * *  :*:.*
```

TLR10 -> TLR3
Y678 -> E789
F672 -> F793
P674 -> A795
K676 -> V797
I678 -> E799
S679 -> L800
C706 -> C827
H707 -> K828
E709 -> K831
F710 -> V832
R741 -> A863

Figure S6. Sequence alignment between the TLR3-TIR domain and the homologous templates. (A) Alignment between the TLR2- and TLR3-TIR domains. (B) Alignment between the TLR6- and TLR3-TIR domains. (C) Alignment between the TLR10- and TLR3-TIR domains. The residues involved in the packing interaction of the dimer are colored red. Residues forming disulfide bonds are colored green. The names of the structural elements comprising the dimer interface are presented at the top of each alignment. Identical residues are indicated with an asterisk (*), residues with strong similarity are indicated with a colon (:), and residues with weakly similar properties are indicated with periods (.). The corresponding key residues in TLR3 are shown in a box to the right of each alignment. The alignments were generated using Clustal-Omega with default parameters.

Table S1. The top-scoring templates used for the homology modeling of TLR3-TIR domains.

PDB code	Sequence identity	e-value	Query coverage
2J67	27.78%	8e-29	99%
4OM7	26.39%	2e-34	100%
1O77	25.00%	2e-29	99%

TLR3-TIR, Toll-like receptor-Toll/interleukin-1 receptor

Table S2. Stereochemical parameters of TLR3 models.

	PROCHECK ([1]Ramachandran plot)			[2]ProSA	[3]Verify 3D	[4]ERRAT
	**Favored region**	**Allowed region**	**Outlier region**			
**[5]S1-TLR3**						
Subunit A	776 (89.5%)	81 (9.3%)	10 (1.2%)	-7.55	89.43%	84.52
Subunit B	784 (90.4%)	77 (8.9%)	6 (0.7%)	-7.49	90.80%	87.60
**[6]S2-TLR3**						
Subunit A	775 (89.4%)	85 (9.8%)	7 (0.8%)	-7.59	92.41%	78.63
Subunit B	782 (90.2%)	76 (8.8%)	9 (1.0%)	-7.2	92.41%	77.72
**[7]S3-TLR3**						
Subunit A	761 (87.9%)	99 (11.4%)	6 (0.7%)	-7.3	87.23%	83.89
Subunit B	790 (91.2%)	69 (8.0%)	7 (0.8%)	-7.1	86.88%	85.18

[1]*Ramachandra plot displays dihedral angles (φ/ψ) for the non*-glycine and -proline *residues of a given protein.*

[2]*ProSA calculates the overall quality score (Z-score) of a given protein structure and indicates its similarity to native proteins in the plot.*

[3]*Verify 3D predicts the compatibility of a given tertiary structure (3D) based on its own amino acid sequence (1D). Usually, a good quality structure possesses a 3D-1D score of >80%.*

[4]*ERRAT calculates the overall quality factor of a given structure based on nonbonded atomic interactions. A good quality model typically obtains a quality factor of >50.*

Table S3. The molecular composition of the simulation systems in the present study.

System	Bilayer	Lipid	Water	Na^+	Cl^-	Ligand	Protein	Duration
S1	POPC	679	98,542	106	N/A	dsRNA	TLR3	200 ns × 3
S2	POPC	677	98,445	106	N/A	dsRNA	TLR3	200 ns × 3
S3	POPC	677	98,278	106	N/A	dsRNA	TLR3	200 ns × 3
S4	POPC	103	4,462	4	N/A	N/A	TLR3-TM	200 ns × 1

POPC, *1-palmitoyl-2-oleoyl-sn-glycero-3-phosphocholine bilayer; dsRNA, double-stranded RNA; TLR3, Toll-like receptor 3; TLR3-TM, NMR structure of TLR3 transmembrane domain*

Table S4. Calculated area per lipid (APL) and thickness of the membrane bilayer in the three molecular dynamics simulation systems.

System	lateral area	Average APL [top leaflet]		Average APL [bottom leaflet]		Thickness
		Without protein	With protein	Without protein	With protein	
S1-TLR3	20840.67 ± 12.42 Å^2	62.58 ± 2.23 Å^2	56.84 ± 3.15 Å^2	60.23 ± 1.96 Å^2	53.45 ± 1.21 Å^2	36.0 ± 2.11 Å
S2-TLR3	21087.41 ± 22.34 Å^2	59.40 ± 2.44 Å^2	55.19 ± 4.24 Å^2	65.48 ± 2.57 Å^2	56.48 ± 2.32 Å^2	37.8 ± 2.45 Å
S3-TLR3	21011.59 ± 12.17 Å^2	61.98 ± 2.27 Å^2	58.36 ± 2.96 Å^2	62.16 ± 2.81 Å^2	53.09 ± 3.01 Å^2	37.9 ± 2.67 Å

Values are averaged over all structural frames in the molecular dynamics trajectories. S1, S2, and S3 represent set 1, 2, and 3, respectively.

Table S5. Intermolecular hydrogen bonds (H-bonds) formed between dsRNA and S1-TLR3.

	TLR3			Occupancy (%)			dsRNA		
	Subunit	Residue	Atom	Run 1	Run 2	Run 3	Atom	Residue	Subunit
Site I	A	N515	HD21	49.35	44.33	43.23	O2P	C24	D
	A	N517	HD22	46.86	43.85	45.83	O2	U22	D
	A	H539	O	34.88	35.87	37.48	HO'2	U22	D
	A	N541	HD22	40.54	43.58	44.55	O2	U21	D
	A	N541	HD21	72.23	74.26	76.62	O2	U22	D
	A	R544	HH21	40.22	46.25	47.25	O2'	C28	C
	A	R544	HH11	53.15	58.14	58.41	O2	C20	D
	B	N515	HD22	38.28	33.23	33.22	O2P	G23	C
	B	N515	HD21	39.96	35.92	35.19	O3'	A22	C
	B	N517	HD21	52.24	54.24	56.22	N3	A21	C
	B	N517	HD22	54.6	53.54	54.1	O2	U27	D
	B	N541	OD1	19.34	16.34	16.53	HO'2	A20	C
	B	R544	HH11	61.63	67.56	67.66	O2'	C19	C
	B	R544	HH21	53.47	54.47	55.44	O2'	C29	D
Site II	A	R64	HE	57.63	53.63	54.64	O3'	G43	D
	A	R64	HH21	56.86	54.48	55.38	O3'	G43	D
	A	R64	HH21	40.22	46.52	46.42	O1P	A44	D
	A	T86	HG1	50.16	54.16	57.15	O2	U5	C
	A	E110	OE2	55.13	56.41	54.12	HO'2	A44	D
	A	E110	OE1	64.42	67.34	63.64	HO'2	A45	D

S1-TLR3, Simulation 1 Toll-like receptor 3; dsRNA, double-stranded RNA. Values presented are averaged over all structural frames in the molecular dynamics trajectories of each set. H-bond occupancy was defined as the fraction of simulation time where an H-bond was formed with cutoff radius of 3.5 Å distance and angle of 30°.

Table S6. Intermolecular hydrogen bonds (H-bonds) formed between dsRNA and S2-TLR3.

	TLR3			Occupancy (%)			dsRNA		
	**Subunit**	**Residue**	**Atom**	**Run 1**	**Run 2**	**Run 3**	**Atom**	**Residue**	**Subunit**
**Site I**	A	N515	HD21	33.67	32.46	34.63	O2P	C24	D
	A	N517	HD21	25.75	29.75	25.72	O4'	C23	D
	A	N517	HD22	16.52	19.54	14.52	O2	U22	D
	A	N541	HD22	17.83	19.84	13.83	O2	U21	D
	A	R544	HH11	48.54	45.55	45.54	O2	C20	D
	A	R544	HH21	33.34	32.34	37.36	O2'	C28	C
	B	N515	HD21	42.65	42.63	62.63	O2P	G23	C
	B	N517	HD22	23.69	20.62	30.64	O2	U27	D
	B	N541	OD1	44.18	47.16	57.17	HO'2	A20	C
	B	N541	HD22	35.36	34.38	64.36	N3	A20	C
	B	R544	HH11	44.97	43.94	43.95	O2'	C19	C
	B	R544	HH11	53.96	57.92	53.95	O2	C19	C
	B	R544	HH21	36.54	38.56	34.53	O2'	C29	C
**Site II**	A	R64	HH21	47.45	43.44	43.46	O1P	A44	D
	A	R64	HH21	58.75	50.37	55.75	O3'	G43	D
	A	F84	O	26.94	25.95	26.93	HO'2	U5	C
	A	E110	OE1	65.53	60.57	63.54	HO'2	A45	D
	B	R64	HH22	43.23	47.38	42.36	O1P	C43	C
	B	T86	HG1	54.52	52.56	53.53	O2'	C6	D
	B	E110	OE1	46.21	48.24	45.42	HO'2	A44	C

S2-TLR3, Simulation 2-Toll-like receptor 3; dsRNA, double-stranded RNA. Values presented are averaged over all structural frames in the molecular dynamics trajectories of each set. H-bond occupancy was defined as the fraction of simulation time where an H-bond was formed with cutoff radius of 3.5 Å distance and angle of 30°.

Table S7. Intermolecular hydrogen bonds (H-bonds) formed between dsRNA and S3-TLR3.

	TLR3			Occupancy (%)			dsRNA		
	**Subunit**	**Residue**	**Atom**	**Run 1**	**Run 2**	**Run 3**	**Atom**	**Residue**	**Subunit**
**Site I**	A	N515	HD21	65.13	66.24	67.65	O2P	C24	D
	A	N517	HD22	46.24	75.25	45.24	O2	U22	D
	A	N541	OD1	17.25	56.25	68.23	HO'2	U21	D
	A	N541	HD22	16.98	65.94	69.93	O2	U21	D
	A	R544	HH21	45.69	44.6	49.64	O2'	C28	C
	A	R544	HH11	34.46	33.44	58.45	O2	C20	D
	B	N515	HD22	64.13	76.16	64.17	O2P	G23	C
	B	N515	HD21	56.36	57.66	56.66	O3'	A22	C
	B	N517	HD22	60.34	65.65	60.94	O2	U27	D
	B	N541	OD1	47.23	66.24	50.23	HO'2	A20	C
	B	N541	HD22	55.23	55.25	55.25	N3	A20	C
	B	R544	HH21	24.14	74.13	63.14	O2'	C29	D
	B	R544	HH11	33.15	76.14	54.13	O2	C19	C
**Site II**	A	R64	HH21	64.53	65.54	55.55	O1P	A44	D
	A	R64	HH21	55.24	51.26	51.27	O3'	G43	D
	A	T86	H	46.26	47.27	63.26	O2'	U5	C
	A	E110	OE2	57.18	55.15	55.15	HO'2	A44	D
	A	E110	OE1	58.71	59.13	61.14	2HO'	A45	D
	B	R64	HH12	60.04	58.03	58.05	O1P	C43	C
	B	R64	HH22	55.95	64.94	44.94	O1P	C43	C
	B	T86	HG1	56.58	78.58	68.18	O2'	C6	D

S3-TLR3, Simulation 3-Toll-like receptor 3; dsRNA, double-stranded RNA. Values presented are averaged over all structural frames in the molecular dynamics trajectories of each set. H-bond occupancy was defined as the fraction of simulation time where an H-bond was formed with cutoff radius of 3.5 Å distance and angle of 30°.

Table S8. Average base-pair parameters of dsRNA molecule in the three molecular dynamics trajectories.

	**Tilt (°)**	**Roll (°)**	**Twist (°)**	**Shift (Å)**	**Slide (Å)**	**Rise (Å)**
Symbol	τ	ρ	ω	D*x*	D*y*	D*z*
S1-dsRNA	-0.79 ± 0.2	7.28 ± 0.45	30.23 ± 1.55	-0.67 ± 0.28	-1.36 ± 0.26	3.27 ± 0.43
S2-dsRNA	-0.52 ± 0.34	8.36 ± 0.96	29.27 ± 1.46	1.27 ± 0.27	-1.21 ± 0.57	3.30 ± 0.48
S3-dsRNA	0.13 ± 0.5	6.24 ± 0.78	29.52 ± 1.2	-0.06 ± 0.01	-1.52 ± 0.36	3.33 ± 0.1
[1]A-DNA	0.1	8.0	31.1	0.0	-1.53	3.32

[1]Standard values obtained for the A-DNA crystal structure. Values presented are averaged over all structural frames in the molecular dynamics trajectories. S1, S2, and S3 represent set 1, 2, and 3, respectively.

Table S9. Average distance between dimer forming residues of the extracellular domains of TLR3.

	Residue	Chain	Cα distance (Å)	Chain	Residue
S1-TLR3-ECD					
	D648	A	11.62 ± 2.92	B	T679
	E652	A	14.0 ± 3.15	B	H682
	T679	A	10.25 ± 2.19	B	D648
	H682	A	10.07 ± 1.34	B	E652
	P680	A	11.92 ± 1.51	B	P680
S2-TLR3-ECD					
	D648	A	9.33 ± 1.46	B	T679
	E652	A	10.85 ± 1.67	B	H682
	T679	A	9.15 ± 1.26	B	D648
	H682	A	10.15 ± 1.53	B	E652
	P680	A	9.39 ± 1	B	P680
S3-TLR3-ECD					
	D648	A	10.57 ± 0.9	B	T679
	E652	A	9.41 ± 0.8	B	H682
	T679	A	11.64 ± 1.15	B	D648
	H682	A	11.83 ± 1.57	B	E652
	P680	A	9.53 ± 0.8	B	P680

Values are averaged over all structural frames in the molecular dynamics trajectories. S1, S2, and S3 represent set 1, 2, and 3, respectively.

Table S10. Selected helical properties of the TM helices in the three TLR3 models.

	Axis length (Å)		Axis tilt (°)		Crossing angle (°)	Axis distance at center (Å)
	TM:A	TM:B	TM:A	TM:B		
S1-TLR3-TM	31.56 ± 2.54	31.25 ± 3.65	26.41 ± 4.04	26.69 ± 4.14	–53.1 ± 3.16	4.52 ± 0.98
S2-TLR3-TM	31.67 ± 2.35	31.13 ± 3.14	20.63 ± 3.54	21.02 ± 3.53	–41.66 ± 2.94	4.43 ± 0.26
S3-TLR3-TM	31.17 ± 2.86	31.07 ± 3.23	30.52 ± 4.51	30.64 ± 4.24	–61.16 ± 3.51	3.60 ± 0.8

Values are averaged over all structural frames in the molecular dynamics trajectories.

Table S11. Comparison of intermolecular hydrogen bonds (H-bonds) formed at the dimerization interface of TIR domains.

	Subunit	Residue	Atom	Occupancy (%)			Atom	Residue	Subunit
				Run 1	Run 2	Run 3			
S1-TLR3-TIR	A	R829	NH1	34.23	34.32	33.22	OE1	E794	B
	A	R829	NH2	45.44	46.44	44.43	OE1	E794	B
	A	Q837	NE2	56.45	55.43	56.45	OE2	E801	B
	A	Q842	NE2	58.8	54.85	55.84	O	E752	B
	A	L864	H	57.73	56.74	53.76	OE1	E755	B
	A	C865	HG	45.55	63.54	45.57	OE1	E755	B
	A	C865	HG	52.16	48.13	54.18	OE2	E755	B
	A	H833	O	53.14	62.14	55.15	H	A802	B
	A	Q838	OE1	32.53	56.55	35.54	ND2	N805	B
	A	H862	O	31.72	46.73	34.73	HZ2	K785	B
S2-TLR3-TIR	A	K765	HZ2	54.64	52.62	53.65	OE1	E790	B
	A	K828	HZ1	87.31	83.22	85.25	O	L822	B
	A	R829	NH1	86.52	84.83	86.78	O	K823	B
	A	R829	NH1	53.42	53.24	55.26	OD1	D824	B
	A	C827	HG	84.32	82.97	84.59	O	K828	B
	A	G796	O	76.53	75.06	77.04	HZ2	K859	B
	A	V797	O	65.67	66.45	64.44	HZ2	K823	B
	A	E801	OE1	54.36	57.44	57.43	ND2	N861	B
	A	D824	OD2	63.77	65.13	63.21	HZ1	K828	B
S3-TLR3-TIR	A	Y764	HH	47.96	55.39	47.96	OE2	E801	B
	A	R829	NH2	66.45	35.43	56.45	OE1	E799	B
	A	E799	OE1	51.74	64.74	41.74	HE	R829	B
	A	C827	O	48.63	46.36	58.36	HZ2	K831	B

TIR, Toll/interleukin-1 receptor; S1, S2, and S3 represent set 1, 2, and 3, respectively. H-bond occupancy was defined as the fraction of simulation time where an H-bond was formed with cutoff radius of 3.5 Å distance and angle of 30°.

Table S12. Intermolecular salt bridges between TIR domain subunits.

	Subunit	Residue	Atom	Distance (Å)	Atom	Residue	Subunit
S1-TLR3-TIR	A	R829	NH1	3.72 ± 1.61	OE1	E794	B
	A	R829	NH1	4.25 ± 1.53	OE2	E794	B
	A	R829	NH2	4.34 ± 1.59	OE1	E794	B
	A	R829	NH2	4.81 ± 1.75	OE2	E794	B
	A	H862	NE2	5.08 ± 2.12	OE2	E778	B
S2-TLR3-TIR	A	K765	NZ	5.41 ± 3.61	OE1	E790	B
	A	K765	NZ	5.41 ± 3.54	OE2	E790	B
	A	H862	NE2	4.31 ± 3.66	OE1	E799	B
	A	R829	NH1	5.77 ± 2.62	OD1	D824	B
	A	R829	NH2	4.11 ± 2.54	OD1	D824	B
	A	D824	OD1	3.33 ± 0.91	NZ	K828	B
	A	D824	OD2	4.59 ± 2.27	NZ	K828	B
S3-TLR3-TIR	A	K765	NZ	9.42 ± 3.37	OE2	E801	B
	A	R829	NH2	3.64 ± 0.89	OE1	E799	B
	A	E799	OE1	6.24 ± 1.81	NE	R829	B
	A	E799	OE1	5.66 ± 2.33	NH1	R829	B

TIR, Toll/interleukin-1 receptor; S1, S2, and S3 represent set 1, 2, and 3, respectively.

Table S13. Buried surface area (BSA) of key residues at the dimer interface of TIR domains.

Subunit	Residue	BSA (Å²)	Subunit	Residue	BSA (Å²)
S1-TLR3-TIR					
A	Q838	142.9	B	Q752	113.6
A	R868	115.6	B	R809	84.3
A	V832	100.9	B	E794	76.0
A	H862	95.9	B	Q753	73.5
A	L864	68.7	B	K785	67.7
A	H833	60.0	B	N805	56.8
A	R829	57.9	B	E755	56.0
A	V836	51.5	B	F793	45.9
A	K831	42.3	B	D792	44.5
A	I840	36.9	B	R749	33.1
A	A863	24.8	B	T751	32.4
A	F798	24.7	B	S783	24.8
A	L800	22.1	B	A802	19.8
A	K828	12.0	B	Y756	18.4
A	G869	11.8	B	F798	17.2
A	A835	11.4	B	K808	16.7
			B	E801	16.1
			B	Q750	13.8
			B	R791	10.3
S2-TLR3-TIR					
A	F798	126.6	B	H862	164.1
A	K828	103.5	B	K823	82.2
A	H833	82.0	B	Y858	70.9
A	D824	58.7	B	V832	61.2
A	Y764	52.0	B	K828	54.5
A	H862	46.8	B	K859	49.2
A	V832	45.0	B	D824	42.1
A	E799	38.1	B	Y764	34.2
A	L800	28.5	B	N861	30.3
A	K823	22.8	B	L864	29.3
A	R829	20.0	B	C827	20.3
A	E790	14.2	B	K765	15.8
A	G796	11.5	B	H833	15.4
A	E789	11.3	B	H819	13.2
S3-TLR3-TIR					
A	Y764	64.3	B	F798	119.3
A	E799	60.9	B	E799	62.6
A	R829	57.6	B	L800	54.4
A	K828	56.5	B	K828	43.3
A	F793	50.1	B	R829	35.4
A	A795	29.1	B	G796	29.0
A	P825	25.1	B	F793	27.2
A	E790	24.6	B	E801	24.7
A	D824	23.8	B	E790	19.5
A	F798	20.8	B	V797	18.5
A	L800	18.2	B	Y764	10.1
A	G796	10.7	B		

Values presented are averaged over all structural frames in the molecular dynamics trajectories of each. S1, S2, and S3 represent set 1, 2, and 3, respectively.
